# Synthesis of 2,1,3-Benzoxadiazole Derivatives as New Fluorophores—Combined Experimental, Optical, Electro, and Theoretical Study

**DOI:** 10.3389/fchem.2020.00360

**Published:** 2020-05-12

**Authors:** Tiago E. A. Frizon, André A. Vieira, Fabricia N. da Silva, Sumbal Saba, Giliandro Farias, Bernardo de Souza, Eduardo Zapp, Michell N. Lôpo, Hugo de C. Braga, Felipe Grillo, Sergio F. Curcio, Thiago Cazati, Jamal Rafique

**Affiliations:** ^1^Department of Energy and Sustainability, Federal University of Santa Catarina, Araranguá, Brazil; ^2^Institute of Chemistry, Federal University of Bahia, Salvador, Brazil; ^3^Center for Natural and Human Sciences-CCNH, Federal University of ABC, Santo André, Brazil; ^4^Chemistry Department, Federal University of Santa Catarina, Florianópolis, Brazil; ^5^Department of Exact Sciences and Education, Federal University of Santa Catarina, Blumenau, Brazil; ^6^Institute of Chemistry, Federal University of Mato Grosso do Sul, Campo Grande, Brazil; ^7^Institute of Science and Technology, Federal University of São Paulo, São José dos Campos, Brazil; ^8^Department of Materials and Metallurgy, Federal Institute of Espírito Santo, Vitória, Brazil; ^9^Physics Department, Federal University of Ouro Preto, Ouro Preto, Brazil

**Keywords:** 2, 1, 3-benzoxadiazole, heterocycles, luminescence, fluorophore, tetrazole

## Abstract

Herein, we report the synthesis and characterization of fluorophores containing a 2,1,3-benzoxadiazole unit associated with a π-conjugated system (D-π-A-π-D). These new fluorophores in solution exhibited an absorption maximum at around ~419 nm (visible region), as expected for electronic transitions of the π-π^*^ type (ε ~2.7 × 10^7^ L mol^−1^ cm^−1^), and strong solvent-dependent fluorescence emission (Φ_FL_ ~0.5) located in the bluish-green region. The Stokes' shift of these compounds is ca. 3,779 cm^−1^, which was attributed to an intramolecular charge transfer (ICT) state. In CHCl_3_ solution, the compounds exhibited longer and shorter lifetimes, which was attributed to the emission of monomeric and aggregated molecules, respectively. Density functional theory was used to model the electronic structure of the compounds **9a–d** in their excited and ground electronic states. The simulated emission spectra are consistent with the experimental results, with different solvents leading to a shift in the emission peak and the attribution of a π-π^*^ state with the characteristics of a charge transfer excitation. The thermal properties were analyzed by thermogravimetric analysis, and a high maximum degradation rate occurred at around 300°C. Electrochemical studies were also performed in order to determine the band gaps of the molecules. The electrochemical band gaps (2.48–2.70 eV) showed strong correlations with the optical band gaps (2.64–2.67 eV).

## Introduction

Conjugated organic compounds, such as benzochalcogenodiazoles, have attracted attention because of their versatility and advantages, including low cost, improved electronic device performance, flexibility, and tuning through structural design (Liu et al., 2015). This class of compounds has been applied in field-effect transistors, sensors, memory devices, and solar cells (Regis et al., 2018). In general, the incorporation of heterocyclic units with electron-donating groups (donors) and electron-accepting groups (acceptors) in the molecular structure increases the conjugation and alters the performance of organic electronic devices (Niu et al., 2007; Fu et al., 2009; Liu et al., 2015).

The derivatives of the 2,1,3-benzothiadiazole (BTD) heterocycle have been extensively investigated due to their recognized photophysical properties, such as intense fluorescence, large Stokes shift, and high extinction coefficient (Gallardo et al., 2012; Wang et al., 2014; Fang et al., 2017; Aguiar et al., 2018). However, there is very limited literature available on the behavior of molecular materials obtained from the 2,1,3-benzoxadiazole (BOX) heterocycle (Pati, 2016). Interestingly, the 7-nitro-1,2,3-benzoxadiazole is generally used as a fluorophore in the design of many analyte-responsive fluorescent probes (Pati, 2016; Wu et al., 2017).

The BOX heterocycle, also known as benzofuran, has a flat molecular and bicyclic conjugated structure, and its derivative compounds with extended conjugates are extremely fluorescent. Because of the presence of an oxygen atom in the heterocycle, BOX is more electronegative when compared to BTD (Bouffard and Swager, 2008; Lin et al., 2011; Zeng et al., 2012; Göker et al., 2014; Regis et al., 2018), and due to this electronegativity, BOX forms coplanar and quinoid structures, giving better stability to the devices manufactured (Liu et al., 2015).

The main applications of BOX are to obtain fluorescent derivatization reagents and use in the detection of heavy metals (Toyo'oka, 2004; Song et al., 2011; Sumiya et al., 2011), in solar cells, and in liquid crystals (Liu et al., 2015; Regis et al., 2018).

As part of our continued involvement in the chemistry of *N*-heterocycles (Saba et al., 2015, 2016, 2017, 2018, 2020; Rafique et al., 2016, 2018a,b; Silva et al., 2017; Bettanin et al., 2018; Peterle et al., 2018; Rodigues et al., 2018; Tornquist et al., 2018; Meirinho et al., 2019; Neto et al., 2020; Scheide et al., 2020) and material sciences (Frizon et al., 2014, 2015, 2016, 2018; Bo et al., 2016, 2018; Westrup et al., 2016; Matzkeit et al., 2018; Regis et al., 2018), herein, we report the synthesis and characterization of BOX derivatives containing electron-donor groups (alkylated tetrazole rings) connected to the 2,1,3-BOX group as an electron-acceptor unit. In addition, thermal, electrochemical, and theoretical predictions were made and photophysical analysis was performed to study the stability, likely geometry, and luminescence properties of these compounds in their ground and excited electronic states.

## Materials and Methods

### Materials and Methods

All reagents were used without further purification and were obtained from commercial sources. The solvents acquired were of commercial grade, with the exception of tetrahydrofuran (HPLC grade), and were purified by methods based on the literature (Armarego and Chai, 2003). The compounds were purified by column chromatography (silica gel) and crystallization from analytical-grade solvents. The reactions were monitored by TLC using Silica Gel 60 (Merck Kieselgel 60F254).

Infrared analysis was performed on an Agilent Cary 600 Series Fourier-transform infrared (FTIR) spectrometer in KBr discs or films. The ^1^H and ^13^C NMR spectra were recorded in chloroform-*d* at 400 and 100 MHz, respectively. The chemical shifts are reported in ppm relative to TMS (0.00 ppm), and the coupling constants are reported in Hz. The chromatograms and mass spectra were acquired on a Shimadzu GCMS-QP5050A spectrometer with a DB5-MS [(5%-phenyl)-dimethylpolysiloxane] capillary column (25 m × 250 μm × 0.25 μm) using an electron ionization voltage of 70 eV. The oven parameters used in the method were 80°C for 6 min, increasing at 15°C min^−1^ to 280°C and remaining at this temperature for 4 min, giving a run time of 23.33 min with an initial delay of 4 min. The gas flow was He at 0.6 mL min^−1^, with pressure S3 of 2.3301 psi. A Varian Cary 50 UV-Vis spectrophotometer was employed to record the absorption spectra. Fluorescence emission and excitation spectra were acquired on a Shimadzu RF5301pc spectrofluorophotometer. The quantum yield (fluorescent fragment length—FFL) was acquired using the dilute optical method. A solution of H_2_SO_4_ (0.5 mol L^−1^) and quinine sulfate (QS) (Φ_FL_ = 0.55) was used as the Φ_FL_ standard (Crosby and Demas, 1971). The uncertainties related to the quantum yield of fluorescence (ΔΦ_FL_) measurements using QS are of the order of 6% (Würth et al., 2011, 2013).

A DropSens BiPotentiostat/Galvanostat (model Stat 400) was used for all electrochemical measurements. The cyclic voltammetry measurements of **9a–d** were obtained in a standard electrochemical cell containing a system of three electrodes: a glassy carbon working electrode, a Pt wire counter electrode, and an Ag/Ag^+^ (AgNO_3_ 0.01 mol L^−1^ in acetonitrile) reference electrode. All measurements were performed in an electrolyte solution [0.1 mol L^−1^ tetra-*n*-butyl ammonium hexafluorophosphate (TBAPF_6_) in CH_2_Cl_2_] purged with purified nitrogen gas. The ferrocene/ferricenium redox pair (Fc/Fc^+^) was used as an internal reference. The spectra for **9a–d** used to estimate the optical band gaps were obtained on a Shimadzu UV-1800 spectrofluorophotometer. Thermogravimetric analysis (TGA) was carried out using a Shimadzu analyzer with the TGA-50 module. A PerkinElmer 2400 Series II Elemental Analyzer System was used to carry out the elemental analysis of the final compounds. The fluorescence decay was obtained through the time-correlated single-photon counting technique, using a @FluoTime200 (PicoQuant) spectrometer. The excitation was performed by a 401-nm pulsed diode laser, applying a repetition rate of 20 MHz. Fluorescence was collected perpendicular to excitation and passed through a polarizer set at the magic angle. The detection system consisted of a monochromator and a microchannel plate photomultiplier (Hamamatsu R3809U-50). Lifetimes were obtained by fitting the fluorescence decays to a convolution of the instrument response function and the sum of exponentials using FluoFit®software. The plots of weighted residuals and reduced chi-squared (χ^2^) were used to accurately determine the quality of the fittings during the analysis procedure. The geometry optimization of **9** was carried out *in vacuo*, using the Orca 4.1 (Neese, 2012) software package and density functional theory (DFT) with the PBE0 functional (Perdew et al., 1996, 1997) and the DEF2-TZVP(-F) basis set for all atoms (Schäfer et al., 1992, 1994; Weigend and Ahlrichs, 2005). Grimme D3 correction was used to include dispersion effects with Becke-Johnson (BJ) damping (Becke and Johnson, 2005; Johnson and Becke, 2005, 2006; Grimme et al., 2010, 2011). In order to accelerate the evaluation of the functionals, the RIJCOSX algorithm was employed, using the resolution of identity approximation for the Coulomb part (RIJ) and the chain of spheres approach for the Fock exchange (COSX) (Izsák and Neese, 2011; Izsák et al., 2013). The vibrational frequencies computed on the optimized geometry of **9** included no imaginary frequencies. In order to simulate the absorption spectra, time-dependent density functional theory (TD-DFT) with the Tamn-Dancoff approximation (TDA) (Petrenko et al., 2011) was employed to obtain the first 10 singlet excited states, using the same protocol. To simulate the emission spectra, the path integral approach implemented by our group in the ORCA_ESD module (Souza et al., 2018, 2019) was employed, using PBE0 to obtain the geometries and Hessians for both the ground and excited states. The line width was set to 450 or 500 cm^−1^, and the default Lorentzian line shape was used to obtain a better fit to the experimental data. The frequencies below 300 cm^−1^ were removed. For the other parameters, the default options were used. Solvent effects were included in the excited-state energies by using the linear response conductor-like polarizable continuum model (LR-CPCM), while the regular CPCM was employed for the ground state (Marenich et al., 2009). Images of the complex geometries were obtained using the Chemcraft program (Chemcraft, 2019).

### Synthesis

#### 2,1,3-Benzoxadiazole-1-oxide (6)

A 500-mL flask was used to obtain a mixture of 2-nitroaniline (9.0 g, 6.5 mmol), tetrabutylammonium bromide (0.3 g, 1.1 mmol), diethyl ether (60 mL), and KOH solution (7 mL, 50% wt). To this mixture, a sodium hypochlorite solution (130 mL, activated chlorine > 10%) was added dropwise. After stirring at room temperature for 7 h, the organic layer was separated, the aqueous layer was extracted with CH_2_Cl_2_ (3 × 1,000 mL), and the combined organic layers were evaporated under reduced pressure. The yellow solid was obtained without further purification. Yield: 89%. m.p. 68°C. IR (KBr) v_max_/cm^−1^: 3,472, 3,100, 3,080, 2,962, 2,935, 2,875, 1,649, 1,614, 1,584, 1,536, 1,484, 1,440, 1,421, 1,382, 1,351, 1,282, 1,200, 1,145, 1,124, 1,012, 963, 928, 890, 833, 743, 734, 671, 633.

#### 2,1,3-Benzoxadiazole (7)

Compound **6** (1.7 g, 13 mmol), triphenylphosphine (4.0 g, 15 mmol), and toluene (150 mL) were placed in a 250 mL flask. The mix was refluxed for 3 h then cooled and filtered. The solvents were evaporated to afford the crude product. The material was chromatographed on silica gel with CH_2_Cl_2_ to afford the 2,1,3-benzoxadiazole **7** as a yellow solid. Yield: 80%. m.p. 69°C. IR (KBr) v_max_/cm^−1^: 3,100, 3,080, 3,010, 2,955, 2,924, 2,853, 2,360, 2,332, 1,969, 1,920, 1,721, 1,650, 1,614, 1,583, 1,535, 1,483, 1,440, 1,422, 1,351, 1,282, 1,263, 1,200, 1,146, 1,125, 1,046, 1,012, 973, 961, 891, 834, 742, 732, 671, 633, 617. ^1^H NMR (400 MHz, CDCl_3_) δ ppm: 7.85 (dd, 2H, *J* = 6.0, 4.0 Hz), 7.41 (dd, 2H, *J* = 6.0, 4.0 Hz). Anal. Calcd. For C_6_H_4_N_2_O [M]^+^ 120.0 found 120.0.

#### 4,7-Dibromo-2,1,3-benzoxadiazole (8)

Compound **7** (1.23 g, 10 mmol) and Fe powder (0.12 g, 2.0 mmol) were placed in a round-bottom flask and heated to 100°C. Br_2_ (1.5 mL, 30 mmol) was added dropwise over 2 h, and then the reaction was refluxed for 3 h. After cooling, the resulting solution was dissolved in CH_2_Cl_2_ (40 mL) and washed with brine (40 mL). The organic fraction was washed with saturated sodium bicarbonate solution (4 × 30 mL), brine (4 × 30 mL), and water (4 × 30 mL). The organic layer was dried and concentrated under vacuum. The brown crude product was chromatographed on silica gel (hexane/ethyl acetate 98:2) to produce **8** as a cream powder. Yield: 40%. m.p. 98–100°C. IR (KBr) v_max_/cm^−1^: 2,923, 2,852, 2,360, 2,342, 1,873, 1,717, 1,695, 1,606, 1,517, 1,493, 1,465, 1,426, 1,378, 1,377, 1,322, 1,300, 1,292, 1,261, 1,204, 1,150, 1,113, 1,085, 107, 1,028, 958, 907, 878, 867, 845, 745, 728, 638, 607. ^1^H NMR (400 MHz, CDCl_3_) δ ppm: 7.51 (s, 2H). GC-MS: RT: 13.92 min.; Calcd. For C_6_H_2_Br_2_N_2_O [M]^+^ 277.8 found 277.9.

#### 5-(4-Bromophenyl)-2*H*-tetrazole (2)

First, 4-Bromobenzonitrile (1.0 g, 5.5 mmol), 20 mL of DMF, ammonium chloride (1.8 g, 33.9 mmol) and NaN_3_ (2.3 g, 35 mmol) were placed in a 100 mL round-bottom flask. The mixture was refluxed for 24 h. At room temperature, the mixture was poured onto ice-water to obtain a precipitate. Then, the mixture was acidified with HCl solution to pH 1. The yellow precipitate was filtered and recrystallized in water/ethanol (1:1). Yield: 90%. IR (KBr) v_max_/cm^−1^: 3,448, 3,090, 3,058, 3,000, 1,905, 1,608. ESI: Anal. Calcd. for C_7_H_5_BrN_4_: m/z 226.9; found m/z 246.9 [M+Na]^+^.

### General Procedure for the Synthesis of Compounds 3a–d

Tetrazole (**2**) (2 mmol), acetone (60 mL), K_2_CO_3_ (2.2 mmol), and 2.2 mmol of the respective alkyl bromide (2-ethylhexyl bromide, octyl bromide, or decyl bromide) were placed in a 250 mL two-neck round-bottom flask. After 45 h of refluxing, the mixture was filtered and concentrated under reduced pressure. The white solid was obtained by recrystallization from ethanol.

#### 5-(4-Bromophenyl)-2-(2-ethylhexyl)-2*H*-tetrazole (3a)

Yield: 72%. ^1^H NMR (400 MHz, CDCl_3_) δ ppm: 8.04 (d, 2H, *J* = 8.8 Hz), 7.63 (d, 2H, *J* = 8.8 Hz), 4.57 (d, 2H, *J* = 6.5 Hz), 2.10 (m, 2H), 1.33 (m, 9H), 0.95 (t, 3H, *J* = 7.04 Hz), 0.89 (t, 3H, *J* = 7.04 Hz). ^13^C NMR (100 MHz, CDCl_3_) δ ppm: 164.1, 132.1, 128.3, 126.6, 124.5, 56.3, 39.8, 30.4, 28.4, 23.8, 22.8, 14.0, 10.5.

#### 5-(4-Bromophenyl)-2-octyl-2*H*-tetrazole (3b)

Yield: 76%. ^1^H NMR (400 MHz, CDCl_3_) δ ppm: 8.04 (d, 2H, *J* = 8.31 Hz), 7.64 (t, 2H, *J* = 8.31 Hz), 4.64 (t, 2H, *J* = 7.34 Hz), 2.06 (m, 2H), 1.37-1.26 (m, 12H), 0.88 (t, 3H, *J* = 7.34 Hz). ^13^C NMR (100 MHz, CDCl_3_) δ ppm: 164.2, 132.1, 128.3, 126.5, 124.6, 53.3, 31.7, 29.4, 28.9, 28.8, 26.4, 22.6, 14.1.

#### 5-(4-Bromophenyl)-2-decyl-2*H*-tetrazole (3c)

Yield: 88%. ^1^H NMR (400 MHz, CDCl_3_) δ ppm: 8.03 (d, 2H, *J* = 8.80 Hz), 7.63 (d, 2H, *J* = 8.80 Hz), 4.64 (t, 2H, *J* = 7.34 Hz), 1.26 (m, 16H), 0.87 (t, 3H, *J* = 6.85 Hz). ^13^C NMR (100 MHz, CDCl_3_) δ ppm: 156.9, 124.8, 121.0, 119.2, 117.2, 46.0, 24.6, 22.3, 22.0, 21.6, 19.0, 15.4, 6.8.

#### 5-(4-Bromophenyl)-2-dodecyl-2*H*-tetrazole (3d)

Yield: 79%. IR (KBr) v_max_/cm^−1^: 2,956, 2,920, 2,849, 1,610. ^1^H NMR (400 MHz, CDCl_3_) δ ppm: 8.03 (d, 2H, *J* = 8.80 Hz), 7.63 (d, 2H, *J* = 8.31 Hz), 4.64 (t, 2H, *J* = 6.85 Hz), 2.06 (m, 2H), 1.37 (m, 2H), 1.26 (m, 16H), 0.89 (t, 3H, *J* = 6.85 Hz). ^13^C NMR (100 MHz, CDCl_3_) δ ppm: 156.8, 124.8, 121.0, 119.2, 117.2, 46.0, 24.6, 22.3, 22.0, 21.6, 19.0, 15.4, 6.8.

### General Procedure for the Synthesis of Compounds 4a–d

#### 4-(4-(2-(2-ethylhexyl)-2*H*-tetrazol-5-yl)phenyl)-2-methylbut-3-yn-2-ol (4a)

In a 125-mL two-neck round-bottom flask containing a desiccant tube of CaCl_2_, a solution was prepared of tetrazole (**3a**) (2.000 g, 5.95 mmol), 60 mL of dry Et_3_N/THF (1:1), bis(triphenylphosphine)palladium dichloride (0.183 g, 0.26 mmol), and triphenylphosphine (0.069 g, 0.26 mmol). After 48 h of reflux, the mixture was vacuum filtered and a white product was obtained by recrystallization from ethanol. Under argon, the mixture was heated to 55°C; then, copper iodide (0.025 g; 0.13 mmol) and 2-methyl-3-butyn-2-ol (0.851 g, 10.11 mmol) in 10 mL Et_3_N/THF (1:1) were added dropwise over 40 min. After 6 h under reflux, the mixture was filtered at room temperature through Celite® and concentrated under reduced pressure. The resulting product was chromatographed on silica gel. For the preparation of compounds **4b**, **4c**, and **4d**, the same methodology was followed, using 5-(4-bromophenyl)-2-octanotetrazole, 5-(4-bromophenyl)−2-decanotetrazol, and 5-(4-bromophenyl)-2-dodecanotetrazol, respectively.

#### 4-(4-(2-(2-Ethylhexyl)-2*H*-tetrazol-5-yl)phenyl)-2-methylbut-3-yn-2-ol (4a)

Yield: 71%. ^1^H NMR (400 MHz, CDCl_3_) δ ppm: 8.1 (d, 2H, *J* = 8.31 Hz), 7.52 (t, 2H, *J* = 8.31 Hz), 4.64 (t, 2H, *J* = 6.85 Hz), 2.51 (s, 1H), 2.11 (m, 1H), 1.65 (s, 6H), 1.33 (m, 8H), 0.95 (t, 3H, *J* = 7.34 Hz), 0.90 (t, 3H, *J* = 6.85 Hz). ^13^C NMR (100 MHz, CDCl_3_) δ ppm: 164.2, 132.1, 128.3, 126.5, 124.6, 53.3, 31.7, 29.4, 28.9, 28.8, 26.4, 22.6, 14.1.

#### 2-Methyl-4-(4-(2-octyl-2*H*-tetrazol-5-yl)phenyl)but-3-yn-2-ol (4b)

Yield: 77%. ^1^H NMR (400 MHz, CDCl_3_) δ ppm: 8.1 (d, 2H, *J* = 8.80 Hz), 7.52 (t, 2H, *J* = 8.31 Hz), 4.64 (t, 2H, *J* = 7.34 Hz), 2.47 (s, 1H), 2.05 (m, 2H), 1.64 (s, 6H), 1.27 (m, 10H), 0.87 (t, 3H, *J* = 6.85 Hz). ^13^C NMR (100 MHz, CDCl_3_) δ ppm: 164.4, 132.1, 127.2, 126.6, 124.7, 95.6, 81.7, 65.6, 53.3, 31.7, 31.5, 29.0, 28.8, 26.4, 22.6, 15.1.

#### 4-(4-(2-Decyl-2*H*-tetrazol-5-yl)phenyl)-2-methylbut-3-yn-2-ol (4c)

Yield: 88%. ^1^H NMR (400 MHz, CDCl_3_) δ ppm: 8.10 (d, 2H, *J* = 8.80 Hz), 7.53 (d, 2H, *J* = 8.80 Hz), 4.65 (t, 2H, *J* = 7.34 Hz), 2.28 (s, 1H), 2.03 (m, 2H), 1.64 (s, 6H), 1.25 (m, 16H), 0.89 (t, 3H, *J* = 6.36 Hz). ^13^C NMR (100 MHz, CDCl_3_) δ ppm: 164.5, 132.2, 127.2, 126.6, 124.7, 95.5, 81.7, 65.6, 53.3, 31.4, 31.5, 29.4, 28.9, 26.4, 22.7, 14.1.

#### 4-(4-(2-Dodecyl-2*H*-tetrazol-5-yl)phenyl)-2-methylbut-3-yn-2-ol (4d)

Yield: 96%. m.p.: 65°C. FTIR (KBr) v_max_/cm^−1^: 3,315, 3,051, 2,956, 2,918, 2,848. ^1^H NMR (400 MHz, CDCl_3_) δ ppm: 8.04 (d, 2H, *J* = 8.80 Hz), 7.64 (d, 2H, *J* = 8.31 Hz), 4.64 (t, 2H, *J* = 6.85 and 7.34 Hz), 2.06 (m, 2H), 1.26 (m, 16H), 0.89 (t, 3H, *J* = 6.85 Hz). ^13^C NMR (100 MHz, CDCl_3_) δ ppm: 164.2, 132.1, 130.2, 127.2, 126.5, 124.6, 53.3, 29.6, 29.4, 26.4, 22.7, 14.1.

### General Procedure for the Synthesis of Compounds 5a–d

Compound (**4a**) (1.61 g, 4.73 mmol), 50 mL of toluene, and NaOH (0.246 g, 6.15 mmol) were combined in a 100 mL round-bottom flask. The solution was distilled while slowly heating, and a mixture of 8 mL toluene/acetone was also distilled. The reaction was monitored by TLC using hexane/ethyl ether (6:4) as eluent. At room temperature, the solution was filtered through Celite® and vacuum-concentrated. The crude solid was purified by column chromatography elution with a solution of hexane/ethyl acetate (95:5), resulting in the desired product at a yield of 90% [40].

#### 2-(2-Ethylhexyl)-5-(4-ethynylphenyl)-2*H*-tetrazole (5a)

Yield: 90%. m.p. 67°C. IR (KBr) v_max_/cm^−1^: 3,291, 2,958, 2,920, 2,842. ^1^H NMR (CDCl_3_, 400 MHz) δ ppm: 8.10 (d, 2H, *J* = 8.31 Hz), 7.52 (d, 2H, *J* = 8.31Hz), 4.57 (d, 2H, *J* = 6.85 Hz), 2.10 (m, 1H), 1.39–1.26 (m, 8H), 0.94 (t, 3H, *J* = 7.34 Hz), 0.89 (t, 3H, *J* = 6.85 Hz). ^13^C NMR (CDCl_3_, 100 MHz) δ ppm: 164.3, 132.1, 127.1, 126.6, 124.6, 95.6, 81.7, 56.2, 39.8, 31.4, 30.3, 28.4, 23.8, 22.8, 13.9, 10.4.

#### 5-(4-Ethynylphenyl)-2-octyl-2*H*-tetrazole (5b)

Yield: 88%. m.p. 65°C. IR (KBr) v_max_/cm^−1^: 3286, 2954, 2916, 2846. ^1^H NMR (CDCl_3_, 400MHz) δ ppm: 8.02 (d, 2H, *J* = 8 Hz), 7.62 (d, 2H, *J* = 8 Hz), 4.64 (t, 2H, *J* = 8 Hz), 2.05 (q, 2H, *J* = 6.8 Hz), 1.41-1.32 (m, 2H), 1.32-1.17 (m, 8H), 0.86 (t, 3H, *J* = 6.46 Hz). ^13^C NMR (CDCl_3_, 100 MHz) δ ppm:164.0, 131.9, 128.1, 126.4, 124.4, 53.2, 31.5, 29.2, 28.7, 26.2, 22.5, 13.9.

#### 2-Decyl-5-(4-ethynylphenyl)-2*H*-tetrazole (5c)

Yield: 87%. m.p. 60°C. IR (KBr) v_max_/cm^−1^: 3,286, 2,954, 2,916, 2,846. ^1^H NMR (CDCl_3_, 400 MHz) δ ppm: 8.02 (d, 2H, *J* = 8 Hz), 7.62 (d, 2H, *J* = 8 Hz), 4.63 (t, 2H, *J* = 6 Hz), 2.05 (q, 2H, *J* = 8 Hz), 1.4-1.32 (m, 2H), 1.40-1.32 (m, 12H), 0.87 (t, 3H, *J* = 6 Hz). ^13^C NMR (CDCl_3_, 100 MHz) δ ppm: 164.1, 132.0, 128.2, 125.4, 124.5, 53.2, 31.8, 29.4, 29.2, 28.8, 26.3, 22.6, 14.0. Anal. Calcd. for C, 73,51%; H, 8.44%; N, 18.05% found C, 72.8%; H, 8.56%; N, 18.27%.

#### 2-Dodecyl-5-(4-ethynylphenyl)-2*H*-tetrazole (5d)

Yield: 88%. m.p. 56°C. IR (KBr) v_max_/cm^−1^: 3,290, 2,950, 2,920, 2,844. ^1^H NMR (400 MHz, CDCl_3_) δ ppm: 8.10 (d, 2H, *J* = 8.22 Hz), 7.53 (d, 2H, *J* = 8.22 Hz), 4.64 (t, 2H, *J* = 7.04 Hz), 2.52 (s, 1H), 2.04 (m, 2H), 1.65 (s, 6H), 1.26 (m, 16H), 0.88 (t, 3H, *J* = 6.46 Hz). ^13^C NMR (100 MHz, CDCl_3_) δ ppm: 164.4, 132.1, 126.6, 124.7, 95.6, 81.7, 65.6, 53.3, 31.9, 31.5, 29.6, 29.3, 29.5, 28.9, 26.3, 22.7, 14.1.

### General Procedure for the Synthesis of Compounds 9a–d

In a 100 mL three-neck round-bottom flask, were mixed compound **8** (0.190 g, 0.68 mmol), 45 mL of dry Et_3_N, bis(triphenylphosphine)palladium (II) dichloride (0.022 g, 0.03 mmol), and triphenylphosphine (0.080 g, 0.30 mmol). After heating to 55°C, copper iodide (0.030 g, 16 mmol) was added. A 15-ml solution of respective aryl acetylene **5a** (0.50 g, 1.47 mmol) in dry Et_3_N was then slowly added dropwise. The reaction remained under reflux for 2 h, being monitored by TLC using a mixture of hexane/ethyl ether (8:2) as the eluent. The mixture was filtered, and the retained solid was dissolved in chloroform and filtered. After concentrating the solvent, a yellow solid was obtained at 71% yield.

#### 4,7-Bis((4-(2-(2-ethylhexyl)-2*H*-tetrazol-5-yl)phenyl)ethynyl)benzo[c][1,2,5] oxadiazole (9a)

Yield: 71 %. m.p. 175°C. IR (KBr) v_max_/cm^−1^: 2,970, 2,951, 2,810, 2,211. ^1^HNMR (400 MHz, CDCl_3_) δ ppm: 8.20 (dd, 4H, *J* = 6.8 Hz, 2.0 Hz), 7.77 (dd, 4 H, *J* = 6.8 Hz, 1.6 Hz), 7.61 (s, 2 H), 4.66 (t, 4H, *J* = 7.2 Hz), 2.07 (q, 4H, *J* = 7.2 Hz), 1.42-1.23 (m, 20H), 0.88 (t, 6H, *J* = 6.8 Hz). ^13^C NMR (100 MHz, CDCl_3_) δ ppm: 164.3, 149.4, 134.4, 132.6, 128.4, 126.9, 123.7, 112.6, 98.8, 85.3, 53.4, 31.7, 29.4, 29.0, 28.9, 26.4, 22.6, 14.1. ESI HRMS: calcd for [C_40_H_44_N_10_O+H]^+^ 681.3772, found 681.3768.

#### 4,7-Bis((4-(2-octyl-2*H*-tetrazol-5-yl)phenyl)ethynyl)benzo[c][1,2,5]oxadiazole (9b)

Yield: 77 %. m.p. 206°C. IR (KBr) v_max_/cm^−1^: 2955, 2914, 2842, 2207. ^1^HNMR (400 MHz, CDCl_3_) δ ppm: 8.21 (dd, 4H, *J* = 6.8, 1.6 Hz), 7.77 (dd, 4H, *J* = 6.8 Hz, 1.6 Hz), 7.64 (s, 2 H), 4.67 (t, 4H, *J* = 7.2 Hz), 2.07 (q, 4H, *J* = 7.2 Hz), 1.40-1.19 (m, 28H), 0.88 (t, 6H, *J* = 6.8 Hz) ppm. ^13^C NMR (100 MHz, CDCl_3_) δ ppm: 164.3, 149.4, 134.4, 132.5, 128.4, 126.8, 123.7, 112.6, 98.7, 85.3, 53.4, 31.9, 29.44, 29.38, 29.34, 29.2, 28.9, 26.4, 22.7, 14.1. ESI HRMS: calcd for [C_44_H_52_N_10_O+H]^+^ 737.4398, found 737.4400.

#### 4,7-Bis((4-(2-decyl-2*H*-tetrazol-5-yl)phenyl)ethynyl)benzo[c][1,2,5]oxadiazole (9c)

Yield: 82 %. m.p. 202°C. IR (KBr) v_max_/cm^−1^: 3,035, 2,968, 2,922, 2,850, 2,202. ^1^HNMR (400 MHz, CDCl_3_) δ ppm: 8.21 (d, 4H, *J* = 8.4 Hz), 7.78 (d, 4H, *J* = 8.4 Hz), 7.28 (s, 2 H), 4.67 (t, 4H, *J* = 7.2 Hz), 2.08 (q, 4H, *J* = 7.2 Hz), 1.41-1.17 (m, 36 H), 0.88 (t, 6H, *J* = 7.2 Hz). ^13^C NMR (100 MHz, CDCl_3_) δ ppm: 164.3, 149.4, 134.4, 132.6, 128.4, 126.8, 123.7, 112.6, 98.7, 85.3, 53.4, 31.9, 29.6, 29.5, 29.4, 29.3, 28.9, 26.4, 22.7, 14.1. ESI HRMS: calcd for [C_48_H_60_N_10_O+H]^+^ 793.5024, found 793.5027.

#### 4,7-Bis((4-(2-dodecyl-2*H*-tetrazol-5-yl)phenyl)ethynyl)benzo[c][1,2,5]oxadiazole (9d)

Yield: 79 %. m.p. 199°C. IR (KBr) v_max_/cm^−1^: 3,031, 2,950, 2,918, 2,850, 2,209. ^1^HNMR (400 MHz, CDCl_3_) δ ppm: 8.21 (d, 4H, *J* = 8.4 Hz), 7.78 (d, 4H, *J* = 8.4 Hz), 7.64 (s, 2 H), 4.59 (d, 4H, *J* = 6.8 Hz), 2.13 (q, 2H, *J* = 6.4 Hz), 1.40-1.27 (m, 16H), 0.97 (t, 6H, *J* = 7.2 Hz), 0.90 (t, 6H, *J* = 6.8 Hz). ^13^C NMR (100 MHz, CDCl_3_) δ ppm: 164.3, 149.4, 134.4, 132.5, 128.4, 126.8, 123.7, 112.6, 98.7, 85.3, 53.4, 31.9, 29.44, 29.38, 29.34, 29.2, 28.9, 26.4, 22.7, 14.1. ESI HRMS: calcd for [C_40_H_44_N_10_O+H]^+^ 681.3772, found 681.3777.

## Results and Discussion

### Synthesis and Spectroscopic Characterization

**Scheme 1:**
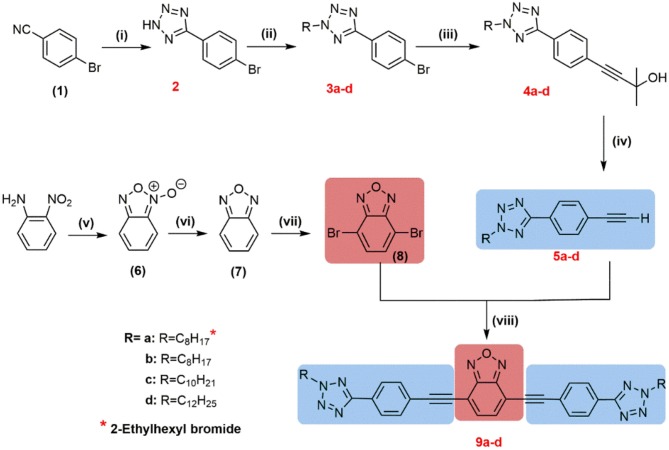
Synthetic route to afford compounds **9a–d**, where (i) NaN_3_, NH_4_Cl, DMF; (ii) butanone, K_2_CO_3_, RBr (R = C_n_H_2n_); (iii) Et_3_N, CuI, PPh_3_, 2-methyl-3-butyne-2-ol, PdCl_2_(PPh_3_)_2_; (iv) KOH, K_3_PO_4_, toluene; (v) TBAB, NaOH, NaClO, diethyl ether; (vi) PPh_3_, toluene; (vii) Br_2_/HBr; (viii) PdCl_2_(PPh_3_)_2_, CuI, triphenylphosphine (C_6_H_5_)_3_P, Et_3_N/THF.

The synthetic route for the preparation of the final desired compounds, based on 1,2,5-benzoxadiazole **9a–d**, is shown in Scheme 1. The synthesis procedure adopted has been described in a previous publication (Frizon et al., 2018). The synthesis starts from the cyclization of 2-nitroaniline with sodium hypochlorite with the catalyst TBAB (tetrabutylammonium bromide) in a basic medium to obtain the *N*-oxide **6**. In sequence, the reduction of the *N*-oxide group using PPh_3_ in xylene gives the heterocycle 2,1,3-benzoxadiazole **7** at 75% yield. In the next stage, the selective bromination reaction of 2,1,3-benzoxadiazole **7** at positions 4 and 7 was performed to obtain the 4,7-dibromo-2,1,3-benzoxadiazole (**8**). The Sonogashira coupling reaction between the aryl dibromide **8** and two equivalents of terminal aryl acetylenes **5a–d**, synthesized previously, allowed the desired compounds **9a–d** to be obtained. After purification in a chromatographic column, the final desired compounds were obtained at good yields (71–82%). All of the final compounds **9a–d** synthesized were characterized by proton and carbon-13 NMR, IR spectroscopy, and mass spectrometry.

### Photophysical Characterization

#### UV-Vis and Fluorescence

The photophysical properties of compounds **9a–d** were determined in chloroform solution at room temperature and are presented in Table 1.

**Table 1:** Photophysical properties of **9a–d** in CHCl_3_ solution.

**Compound**	**$\lambda_{\text{Abs}}^{\max}/{\text{n}}^{\text{m}}$^a^ (ε/10^**4**^ mol L^**−1**^ cm^**−1**^)^b^**	**$\lambda_{\text{FL}}^{\max}/{\text{nm}}^{\text{a,c}}$**	**Stokes Shift/cm^-1^^a^**	**Φ$\varphi_{\text{FL}}^{\text{d}}$**	**$\tau_{\text{fe}}^{/}\text{ns}$**
**9a**	417 (3.0)	494	3,738	0.54	2.73
**9b**	419 (2.7)	498	3,786	0.52	2.68
**9c**	417 (3.6)	495	3,779	0.52	2.68
**9d**	419 (4.2)	498	3,786	0.51	2.68

a *L mol^-1^ cm^-1^*.

b *In chloroform solution (1.0 × 10^-5^ mol L^-1^)*.

c *Excited at absorption maxima*.

^d^*Quantum yields were determined with the quinine sulfate solution as reference (Φ_F_ = 0.55 in 0.5 mol L^−1^ H_2_SO_4_)*.

^e^*Average lifetimes were calculated using τ_f_ = $\Sigma \tau_{\text{i}}^{2}$A_i_/Στ_i_A_i_ due to multi-exponential profile. Fluorescence decays were measured at maxima of the emission intensities of the compounds after excitation at 401 nm*.

**Figure 1:**
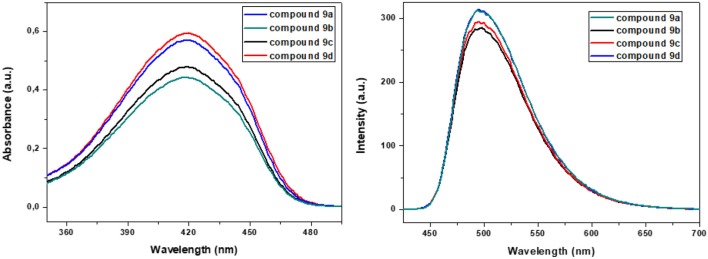
Absorbance (**left**) and emission (**right**) spectra for compounds **9a–d** in CHCl_3_ solution (10^−5^ mol L^−1^).

The absorption and emission spectra obtained for **9a–d** in chloroform solution are shown in Figure 1. All compounds displayed similar absorption spectra with maxima at 419 nm, which are attributed to a π-π^*^ transition and showed high molar absorption coefficients (~3.4 × 10^−4^ L mol^−1^ cm^−1^), which is characteristic of the benzoxadiazole heterocycle. Also, after excitation at the lower energy band, all compounds showed a strong bluish-green fluorescence emission in solution (λ_FL_ = 450–650 nm), with emission maxima ranging from 494 to 498 nm. The values for the Stokes shifts for compounds **9a–d** are in the range of 3,738–3,786 cm^−1^, indicating a possible intramolecular charge transfer (ICT) in the excited state (Neto et al., 2007). The fluorescence quantum yields (Φ_FL_) of **9a–d** were determined in chloroform using quinine sulfate as the standard (Φ_*r*_ = 0.55 in 0.5 mol L^−1^ H_2_SO_4_). The Φ_FL_ values could be calculated using the equation (Fery-Forgues and Lavabre, 1999): Φ_*FL*_ = Φ_*r*_ (F_s_/F_r_)(A_r_/A_s_)(η_s_/η_r_), where s and r denote the sample and reference, respectively, η is the solvent refractive index, F is the integrated fluorescence intensity, and A is the value of absorbance at the excitation wavelength used to record the fluorescence spectrum. The values obtained are summarized in Table 1, where it can be noted that all compounds presented quantum yields (Φ_*FL*_) of 0.51 to 0.54. The values obtained for compounds **9a–d** are considerably higher than those reported in our previous articles for compounds containing the heterocycles 2,1,3-benzoselenadiazole (Regis et al., 2018) and 2,1,3-benzotiadiazole (Frizon et al., 2016).

The singlet excited-state lifetimes of compounds **9a–d** in CHCl_3_ solvent were investigated by time-resolved fluorescence spectroscopy, using a wavelength of 401 nm for the excitation. Compounds **9a–d** were evaluated in chloroform solution because of the higher affinity of this solvent (due to the alkyl chains) with the aromatic center. The singlet excited-state average lifetimes for compounds **9a–d** are shown in Table 1, and their values are similar. All fluorescence decays at emission maxima of compounds **9a–d** (see Figure S1) were best fitted by a bi-exponential function. The excited-state lifetimes and their relative amplitudes for **9a–d** in chloroform solution are shown in Table S1 (see **ESI**). The longer lifetimes (τ_1_) were attributed here to the emission of monomeric **9a–d**, while the shorter lifetimes (τ_2_) were attributed to the emission of **9a–d** aggregated in chloroform solution, even at 10^−5^ mol L^−1^, since the molecular aggregation could reduce the excited-state lifetimes due to the enhanced radiationless path of the excited-state deactivation. With the exception of **9a**, the relative amplitudes of aggregate emissions (A_2_) were higher than 70% in dilute chloroform solution (see Figure S1). The relative amplitude A_2_ of **9a** was close to 50%, showing the possible influence of the branched chain in forming fewer aggregates than the linear chain. A hypothesis of the aggregation of **9a–d** in chloroform solution is proposed based on the fluorescence excitation measurements. The fluorescence excitation spectra for **9a–d** in chloroform solution are shown in Figure S2 and compared with the absorption spectra. The excitation spectra for the compounds show a different vibrational progression and are red-shifted when compared to the absorption spectra, suggesting aggregation, since the excitation spectrum should be identical in shape to the absorption spectrum, provided that there is a single species in the ground state. In contrast, the excitation and absorption spectra are no longer superimposable when species exist in different forms, such as aggregates, in the ground state (Valeur, 2012). When compared with similar benzothiadiazole derivatives previously reported by our group (Westrup et al., 2016), the benzoxadiazole-based compounds **9a–d** showed a greater tendency toward aggregate formation. This could be associated with the benzoxadiazole moiety and the higher dipole moment of this heterocycle (Tobiason et al., 1973).

It was observed that the size of the alkyl chain did not significantly influence the deactivation of the excited states, suggesting that only the conjugated aromatic part contributes to the frontier orbitals (HOMO and LUMO), which are responsible for the radiative processes.

#### Solvatochromism

**Figure 2:**
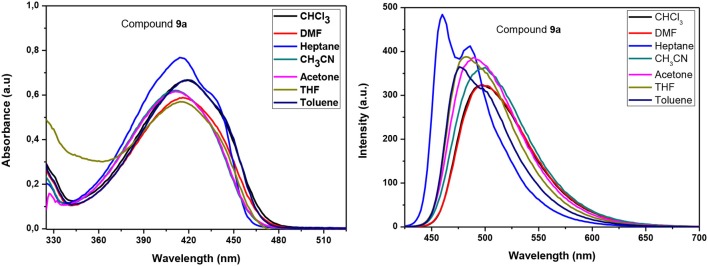
Absorbance (**left**) and emission (**right**) spectra for **9a** in different solvents.

A solvatochromic study of compounds **9a–d** was performed to ascertain the role of solvent polarity in the absorption and fluorescence emission. The UV-vis absorption and fluorescence properties were measured in heptane (Hep), toluene (Tol), chloroform (CHCl_3_), tetrahydrofuran (THF), acetone (Acet), *N, N*-dimethylformamide (DMF), and acetonitrile (CH_3_CN) solutions. The UV-Vis absorption and emission spectra for **9a** in various solvents are shown in Figure 2, and the specifics of the absorption and fluorescence emission of these compounds are listed in Table 2.

**Table 2:** The absorption and emission wavelengths, molar absorption coefficient, and Stokes shifts of compound **9a** in various solvents.

**Compound**	**Absorption^b^ λ_max_(nm) (ε)^a^**	**Emission^c^ λ_max_ (nm)**	**Stokes shift (cm^**−1**^)**
**9a** (Hept)	416 (2.7 × 10^7^)	487	3,505
**9a** (Tol)	420 (2.8 × 10^7^)	477	2,845
**9a** (CHCl_3_)	420 (2.8 × 10^7^)	499	3,769
**9a** (THF)	416 (2.7 × 10^7^)	484	3,377
**9a** (Acet)	413 (2.7 × 10^7^)	491	3,846
**9a** (DMF)	417 (2.8 × 10^7^)	499	3,941
**9a** (CH_3_CN)	413 (2.7 × 10^7^)	499	4,173

a *L mol^-1^ cm^-1^*.

b *[c] = 1.5 × 10^-5^ mol L^-1^*.

c *Excited at absorption maxima*.

With increasing solvent polarity, the absorption bands of compound **9a** showed a slight red-shift, indicating a small solvatochromic effect on the ground state. The fluorescence spectra showed a notable hypsochromic shift of the maximum emission in heptane, toluene, and THF (less polar solvents) and higher vibrational resolution when compared to CHCl_3_. The largest hypsochromic shifts were observed for THF and toluene (15 and 22 nm, respectively). This type of optical behavior in apolar organic solvents is similar to that exhibited by benzothiadiazole (Westrup et al., 2016). With the use of a more polar solvent like *N,N*-dimethylformamide or acetonitrile, a small hypsochromic shift in the emission maxima compared to CHCl_3_ was also registered. This solvatochromism behavior is observed in molecules exhibiting larger dipole moments and charge transfer characteristics in the excited state, that is, an ICT state (Liang et al., 2015).

The excited-state lifetimes and relative amplitudes of **9a** at 10^−5^ mol L^−1^ in heptane, toluene, tetrahydrofuran, and acetone are shown in Table S2. In all solvents, **9a** exhibited two excited-state lifetimes. Except in heptane, the longer excited-state lifetimes (τ_1_) were over 3 ns and the shorter lifetimes (τ_2_) ranged from 2.2 to 1.8 ns. In addition, the shortest lifetime dominates the fluorescence (A_2_) of compound **9a**. The longer excited-state lifetimes (τ_1_) were attributed here to monomeric emission of **9a**, while the shorter excited-state lifetimes (τ_2_) could be associated with the emission provided by aggregates of molecules **9a** in toluene, tetrahydrofuran (THF), and acetone solutions. In heptane solution, the longest lifetime (τ_1_) of molecule **9a** was found to be 2.1 ns and the shortest lifetime (τ_2_) around 1.4 ns, which could be related to the poor stabilization of the excited state by the non-polar solvent. In addition, the contributions of the longest lifetime (A_1_) and the shortest lifetime (A_2_) to the emission of **9a** in heptane were similar, suggesting that the emission of **9a** in heptane solution is provided equally by the monomer and the aggregate of **9a**.

The quantum yields of **9a** in different solvents are shown in Table S2. The quantum yields increase with increasing affinity of the solvent for compound **9a**. The quantum yields for **9a** were 0.27 in heptane solution and 0.71 in acetone solution. In toluene, THF, and acetone solutions, the emission lifetime results suggest that the aggregate dominates the emission of compound **9a**; however, monomeric emission of **9a** also contributes to the fluorescence, mainly in acetone solution. As the ICT state is known to be able to non-radiatively deactivate excited species, the quantum yield results are in agreement with the proposed molecular aggregation, which suppresses the ICT in the excited state of the monomer and increases the quantum yield in polar solvents (Liang et al., 2015).

#### Theoretical Modeling of the Electronic Structure and Fluorescence

**Figure 3:**
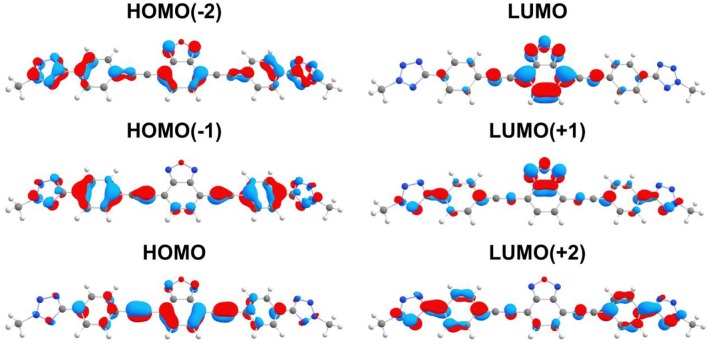
Frontier molecular orbitals of **9** calculated using PBE0/def2-TZVP(-f).

To gain a better understanding of the absorption and luminescence properties of **9a–d**, we carried out calculations using DFT for a representative molecule without the aliphatic carbon chains (**9**). The calculated frontier orbitals are shown in Figure 3. It can be observed that the HOMO has major contributions from the benzene ring of the benzoxadiazole moiety and from the triple bonds, whereas the LUMO is mostly a π^*^ orbital delocalized over the benzoxadiazole. The HOMO (−2) and HOMO (−1) are more evenly spread through the molecule, while the LUMO (+1) is more centered in the benzoxadiazole ring and LUMO (+2) in the benzene rings close to the tetrazole.

**Figure 4:**
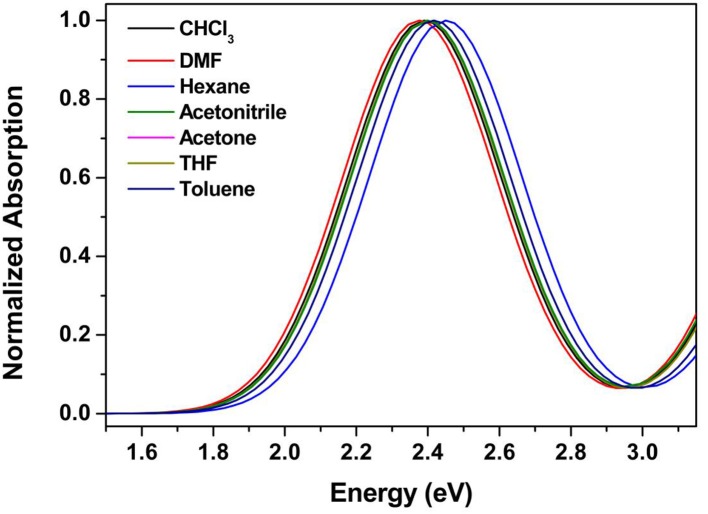
Theoretical absorption spectra calculated in different solvents using PBE0/def2-TZVP(-f) and convoluted with Gaussians of 0.25 eV width.

TD-DFT calculations were performed to acquire information related to the excited states. The absorption spectra were calculated and convoluted with Gaussians, reproducing the experimental spectra, as shown for each molecule in Figure 4. Table 3 shows some of the first two calculated transitions, which were observed in the experimental spectra. To simulate the solvent dependence, the linear response CPCM was used to account for the effect of the solvent on the energy. The two transitions predicted for **9** correspond to those observed in the measured spectra, and, in all solvents, the lowest energy transition originates from a HOMO → LUMO (95) excitation. The calculated spectra showed the same solvent-dependence observed in the UV-Vis spectra. With increasing solvent polarity, the first excited state is predicted to be stabilized and the transition matrix element of the dipole moment operator decreases. These results, together with an analysis of the molecular orbitals involved in the first electronic transition, lead to the assignment of a π-π^*^ transition with the characteristics of a charge transfer excitation, thus explaining the large transition dipole moment and Stokes shift observed.

**Table 3:** Data for the TD-DFT excitations using PBE0/def2-TZVP(-f).

	**Energy**				
**State**	**eV**	**Solvent shift**	***f***	**| < I|μ|F>|^**2**^**	**Attribution^a^**
**Hexane**					
S_1_	2.45	0.14	1.7804	29.67	H → L (95)
S_2_	3.04	0.19	0.0002	0.004	H-1 → L (88)
**Toluene**					
S_1_	2.42	0.18	1.7323	29.25	H → L (95)
S_2_	3.00	0.23	0.0003	0.004	H-1 → L (88)
**CHCl**_**3**_					
S_1_	2.40	0.18	1.6547	28.19	H → L (95)
S_2_	2.98	0.23	0.0003	0.004	H-1 → L (88)
**THF**					
S_1_	2.40	0.17	1.6347	27.83	H → L (95)
S_2_	2.99	0.22	0.0003	0.004	H-1 → L (88)
**Acetone**					
S_1_	2.40	0.16	1.6038	27.29	H → L (95)
S_2_	2.99	0.21	0.0003	0.004	H-1 → L (89)
**DMF**					
S_1_	2.38	0.18	1.5804	27.15	H → L (95)
S_2_	2.95	0.90	0.0037	0.0521	H-3 → L (94)
**Acetonitrile**					
S_1_	2.40	0.16	1.5962	27.16	H → L (95)
S_2_	3.00	0.21	0.0003	0.004	H-1 → L (89)

a *Transitions with high percentage contributions are shown in parentheses*.

**Figure 5:**
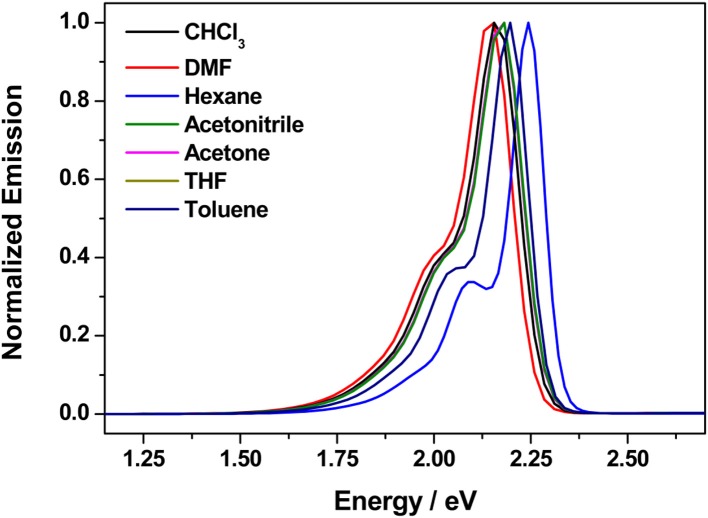
Predicted emission spectra calculated in different solvents using PBE0/def2-TZVP(-f).

The fluorescence spectra were also calculated for **9** using the path integral approach developed by our group (Souza et al., 2018, 2019). The PBE0 functional was chosen to obtain the adiabatic electronic energy differences. The emission spectra, shown in Figure 5, are also consistent with the experimental results. The emission peak was shifted for the different solvents; for example, hexane and toluene showed emission at higher energy compared with the other solvents. The main vibrational progression observed in the experimental spectra of non-polar solvents is due to the C=C and C=N stretching modes, with the energy separation of these peaks beginning at around 0.15 eV (1,200 cm^−1^).

### Thermal Properties

**Figure 6:**
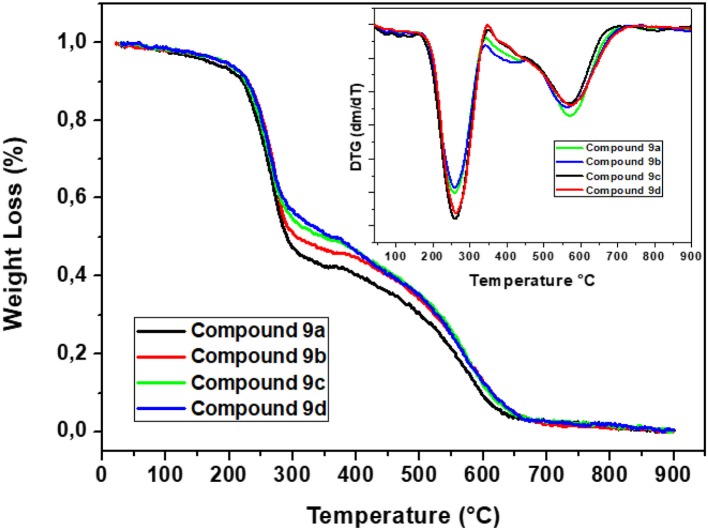
Thermograms (TGA) and DTG curves of compounds **9a–d**.

The thermal properties of compounds **9a–d** were obtained by thermogravimetric analysis (TGA) (Figure 6). The DTG curves for the final compounds were processed from the TGA curves. The complete degradation process indicates a mass loss of around 100%. Three thermal events were observed for these compounds, corresponding to a mass loss of almost 55% at an initial decomposition temperature of 220°C, with a maximum degradation rate observed at around 300°C. The second thermal event was detected at approximately 305°C, and this event is related to a loss of 51%. A small thermal event was detected at ~650°C, and this event is related to a loss of 4%.

### Electrochemical Characterization

**Figure 7:**
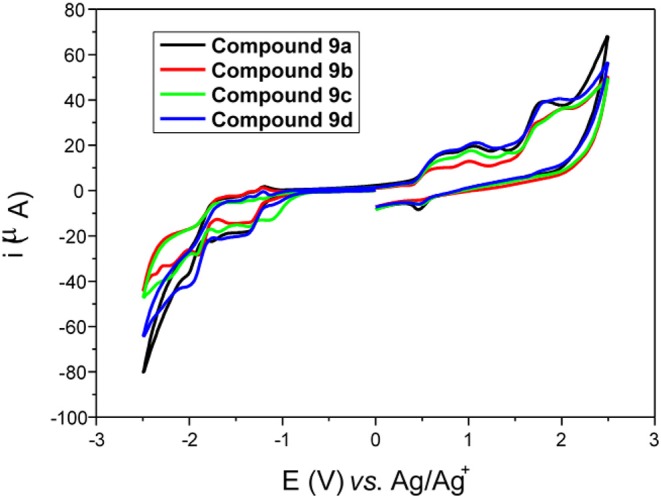
Cyclic voltammogram of compounds **9a–d** on a glassy carbon electrode in 0.1 TBAPF_6_/CH_2_Cl_2_ at 100 mV s^−1^.

Compounds **9a–d** show very distinct reduction processes in the negative scan (Figure 7). The first may be associated with a quasi-reversible reduction of the benzoxadiazole, indicating that the benzochalcogen ring is reduced in preference to the triple bond. Benzochalcogen is a well-known heteroatomic compound with a high electron-accepting part, because it has two electron-withdrawing imine groups (C=N) (Omer et al., 2009).

Thus, in the case of **9a–d**, this peak can be attributed to the benzoxadiazole group and the reduction peak can probably be assigned to the anion radical species. The second process that occurs at much more cathodic values may be associated with an irreversible electrochemical process with triple bond reduction (Neto et al., 2005, 2013). In the positive scan, various oxidation processes were observed, which probably include oxidation of the tetrazole portion of the molecule, and this process could involve the formation of an intermediate radical cation species.

The energies of HOMO and LUMO boundary orbitals of π-conjugated systems (D-π-A-π-D), such as benzochalcogenic derivatives, can be defined by the electron affinity (EA) and ionization potential (IP). These can be accessed by the evaluation of electron affinity (EA) and the ionization potential (IP), based on the values of reduction and oxidation potentials obtained by electrochemical assays (Neto et al., 2013; Frizon et al., 2014). The EA and IP were determined using the following empirical formula (Neto et al., 2005): EA = E${}_{\text{red}}^{\text{onset}}$ + 4.44 eV and IP = E${}_{\text{oxi}}^{\text{onset}}$ + 4.44 eV, where E${}_{\text{red}}^{\text{onset}}$ and E${}_{\text{oxi}}^{\text{onset}}$ are the onset potentials of reduction and oxidation, respectively. These onset potentials were corrected from Ag/Ag^+^ to NHE using the Fc/Fc^+^ pair. Analyses of the HOMO-LUMO energy level calculations of **9a–d** were performed from benzoxadiazole potentials. The band gap values obtained by cyclic voltammetry and UV-vis spectroscopy showed good agreement between the results obtained with the two different techniques. The results of the electrochemical assays conducted with **9a–d** showed that changes in the length of the alkyl group attached to the tetrazole ring did not significantly influence the redox potentials of the molecules. The $E_{gap}^{ele}$ values obtained for molecules **9a-d** were higher than those for benzoselenadiazole (Regis et al., 2018) and benzothiadiazole (Frizon et al., 2016). This change to more positive values, obtained due to the addition of a heavier chalcogen atom to the heterocycle, can be attributed to an increase in the electronegativity of the chalcogens and proved to be a relevant factor in determining the energies of HOMO and LUMO (Kawashima et al., 2015; Ghosh et al., 2018).

The electrochemical data and optical band gaps obtained for compounds **9a–d** are summarized in Table 4.

**Table 4:** Optical and electrochemical properties of compounds **9a–d**, where $E_{oxi}^{onset}$ is the onset potential of oxidation, $E_{red}^{onset}$ is the onset potential of reduction, I_p_ (HOMO) is the ionization potential, E_a_ (LUMO) is the electron affinity, E_g_ is the band gap, and λ_onset_ is the absorption onset wavelength.

**Parameter**	**Compound**			
	**9a**	**9b**	**9c**	**9d**
$E_{oxi}^{onset}$ (V)^a^	1.964	1.934	2.001	1.953
$E_{red}^{onset}$ (V)^a^	−0.726	−0.756	−0.476	−0.746
IP (HOMO) (eV)^b^	−6.404	−6.374	−6.441	−6.393
EA (LUMO) (eV)^c^	−3.714	−3.684	−3.994	−3.964
$E_{gap}^{ele}$(eV)	2.69	2.69	2.48	2.70
λ_onset_ (nm)	467.1	468.9	468.8	468.1
$E_{gap}^{opt}$ (eV)^d^	2.65	2.64	2.64	2.65

a *vs. NHE*;

b *IP= –(E_ox_+ 4,44) eV*;

c *EA= –(E_red_+ 4,44) eV*;

d *Optical bandgap calculated on the low energy band edge of the absorption spectrum ($E_{gap}^{op}=$ 1240/λ_onset_)*.

## Conclusions

In this study, a new series of fluorophores, with D-π-A-π-D molecular architectures, based on 2,1,3-benzoxadiazole derivatives, was synthesized and characterized. The photophysical behavior of the compounds was evaluated by combining the following spectroscopic techniques: UV-vis absorption spectroscopy, stationary fluorescence spectroscopy, and time-resolved spectroscopy. The series of molecules in solution exhibited, in the visible region, a maximum absorption (~419 nm) that can be attributed to electronic transitions of the type π-π^*^ (ε ~3.4 × 10^−4^ L mol^−1^cm^−1^). Compounds **9a–d** exhibited intense fluorescence (Φ_FL_ ~0.5) located in the bluish-green region (494–498 nm) with a solvent polarity dependence resulting in a large Stokes shift (ca. 3,779 cm^−1^), which was associated with an ICT in the excited state. The data obtained from DFT and TD-DFT indicate that the ground and first excited states are a π-type orbital. Therefore, a π-π^*^ transition with the characteristics of a charge transfer excitation can be assumed, confirming the ICT state. Compounds **9a–d** showed a strong tendency to form aggregates even in dilute solution, exhibiting in CHCl_3_ solution longer and shorter lifetimes, on the nanosecond timescale, which were here attributed to the emission of monomeric and aggregated molecules, respectively. The thermal characterization allowed the thermal stability of compounds **9a–d** to be evaluated, and a high maximum degradation rate was observed at around 300°C. Small electrochemical band gap (2.48 to 2.70 eV) and optical band gap (2.64 to 2.67 eV) values were obtained for compounds **9a–d**, these being in agreement with results for similar compounds previously reported in the literature. The redox potentials of **9a–d** did not change significantly with variations in the length of the alkyl group attached to the tetrazole ring. The recognition of the structure–property relationship of benzoxadiazole could guide further studies in the search for luminescent materials with better performance than benzothiadiazole.

## Data Availability Statement

All datasets generated for this study are included in the article/Supplementary Material.

## Author Contributions

TF, AV, and JR conceived the project and wrote the manuscript. TF, JR, AV, SS, and ML performed the synthesis and characterization. AV, FS, TF, and TC studied the photophysical properties (absorption and fluorescence spectra). HB, FG, and TF studied the thermal properties. EZ performed the electrochemical studies. GF and BS made the theoretical predictions. SC and TC studied the singlet excited-state lifetimes. All authors discussed the results and commented on the manuscript.

## Conflict of Interest

The authors declare that the research was conducted in the absence of any commercial or financial relationships that could be construed as a potential conflict of interest.

## Supplementary Material

For compounds **9a–d**, the absorption (Abs) and fluorescence excitation (Ex) spectra, NMR spectra, FTIR spectra, and HRMS are available in Figures S3–S23.
