# Supplementary material for: Synthesis of 2,1,3-Benzoxadiazole Derivatives as New Fluorophores—Combined Experimental, Optical, Electro, and Theoretical Study
Source: Front Chem. 2020 May 12;8:360. doi: 10.3389/fchem.2020.00360 (PMC7235381; doi:10.3389/fchem.2020.00360)
Supplement: Supplementary file 1 [file Data_Sheet_1.pdf]

Supplementary Material

Synthesis of 2,1,3-Benzoxadiazole Derivatives as New Fluorophores—Combined Experimental, Optical, Electro, and Theoretical Study

Tiago E. A. Frizon[1]*, André A. Vieira[2]*, Fabricia N. da Silva[2], Sumbal Saba[3]*, Giliandro Farias[4], Bernardo de Souza[4], Eduardo Zapp[5], Michell N. Lôpo[6], Hugo de C. Braga[7], Felipe Grillo[8], Sergio F. Curcio[9], Thiago Cazati[9], Jamal Rafique[6]*

[1] Department of Energy and Sustainability, Federal University of Santa Catarina, Araranguá, Brazil.

[2] Institute of Chemistry, Federal University of Bahia, Salvador, Brazil.

[3] Center for Natural and Human Sciences-CCNH, Federal University of ABC, Santo André, Brazil,

[4] Chemistry Department, Federal University of Santa Catarina, Florianópolis, Brazil.

[5] Department of Exact Sciences and Education, Federal University of Santa Catarina, Blumenau, Brazil.

[6] Institute of Chemistry, Federal University of Mato Grosso do Sul, Campo Grande, Brazil.

[7] Institute of Science and Technology, Federal University of São Paulo, São José dos Campos, Brazil.

[8] Department of Materials and Metallurgy, Federal Institute of Espírito Santo, Vitória, Brazil.

[9] Physics Department, Federal University of Ouro Preto, Ouro Preto, Brazil.

E-mail: tiago.frizon@ufsc.br (T.E.A. Frizon), vieira.andre@ufba.br (A.A. Vieira),

sumbal6s@gmail.com; sumbal.saba@ufabc.edu.br (S. Saba)

jamal.chm@gmail.com; jamal.rafique@ufms.br (J. Rafique)

Table of Contents

1. Table……………………………………………………………………..…… 3

2. Fluorescence decay curves ………………………………………………… 4

3. Absorption (Abs) and fluorescence excitation (Ex)……………………….……. 5

4. NMR spectra... 7

5. Infrared spectra... 11

6. HRMS …………………………………………………..………………… 13

1. TABLE:

Table 1S: Excited state lifetimes and their relative amplitudes at emission maxima of **9a-d** in chloroform solution

Compounds	τ_1 / ns	A_1 / %	τ_2 /ns	A_2 / %	χ^2	λ^{max}_{FL}/nm
**9a**	3.28±0.04	47.85	2.23±0.04	52.15	0.970	494
**9b**	3.61±0.05	29.90	2.28±0.03	70.10	0.983	498
**9c**	3.85±0.06	23.65	2.31±0.03	76.35	0.981	495
**9d**	3.95±0.07	21.91	2.32±0.03	78.09	1.005	498

Table 2S: Fluorescence properties of compound **9a** at 10^{-5}M in heptane, toluene, tetrahydrofuran (THF) and acetone solutions

Solvents	τ_1 /ns	A_1 /%	τ_2 / ns	A_2 / %	χ^2	$<\tau_f>^a$ /ns	$\lambda^{b,c}_{em}$ / nm	Φ_{FL}^d
**Heptane**	2.11±0.03	47.76	1.44±0.03	52.24	0.968	1.80	457	0.27
**Toluene**	3.27±0.08	11.68	1.85±0.02	88.32	1.084	2.12	474	0.44
**THF**	3.37±0.06	18.80	1.97±0.02	81.20	1.006	2.42	478	0.53
**Acetone**	3.88±0.07	25.71	2.20±0.04	74.29	1.030	2.84	486	0.71

[a]Average lifetimes were calculated using $<\tau_f> = \Sigma\tau_i^2A_i/\Sigma\tau_iA_i$; [b]Excited at maximum absorption; [c]Maxima emission wavelength; [d]Relative quantum yields in solution determined using quinine sulfate as standard (Φ_{Fl} = 0.546 in 1N H_2SO_4);

2. FLUORESCENCE DECAY CURVES:

Figure 1S: Fluorescence decay curves for compounds **9a-d** in chloroform solution (10^{-5} mol L^{-1}) using a 401 nm excitation wavelength recorded at emission maxima fitted by a bi-exponential function

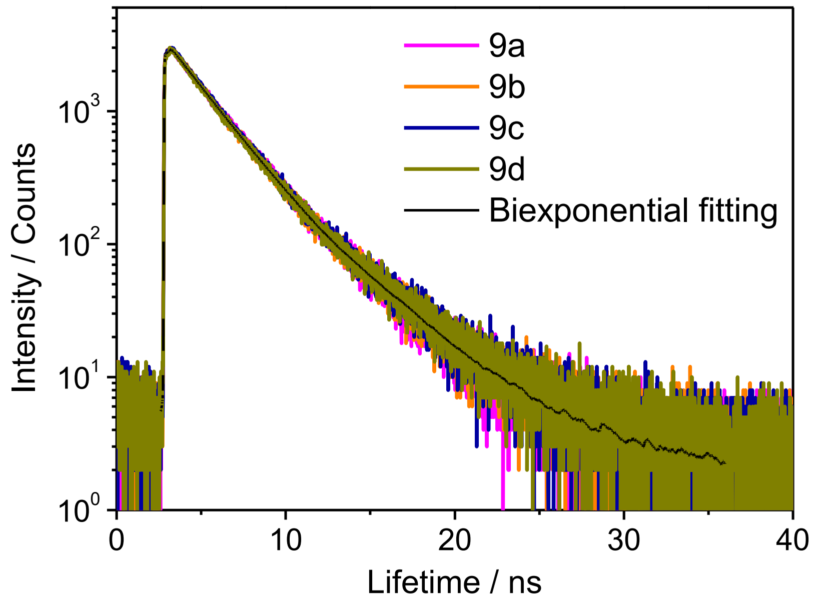

3. ABSORPTION (ABS) AND FLUORESCENCE EXCITATION (EX)

Figure 2S: Absorption (Abs) and fluorescence excitation (Ex) spectra of compounds **9a-d** in chloroform solution

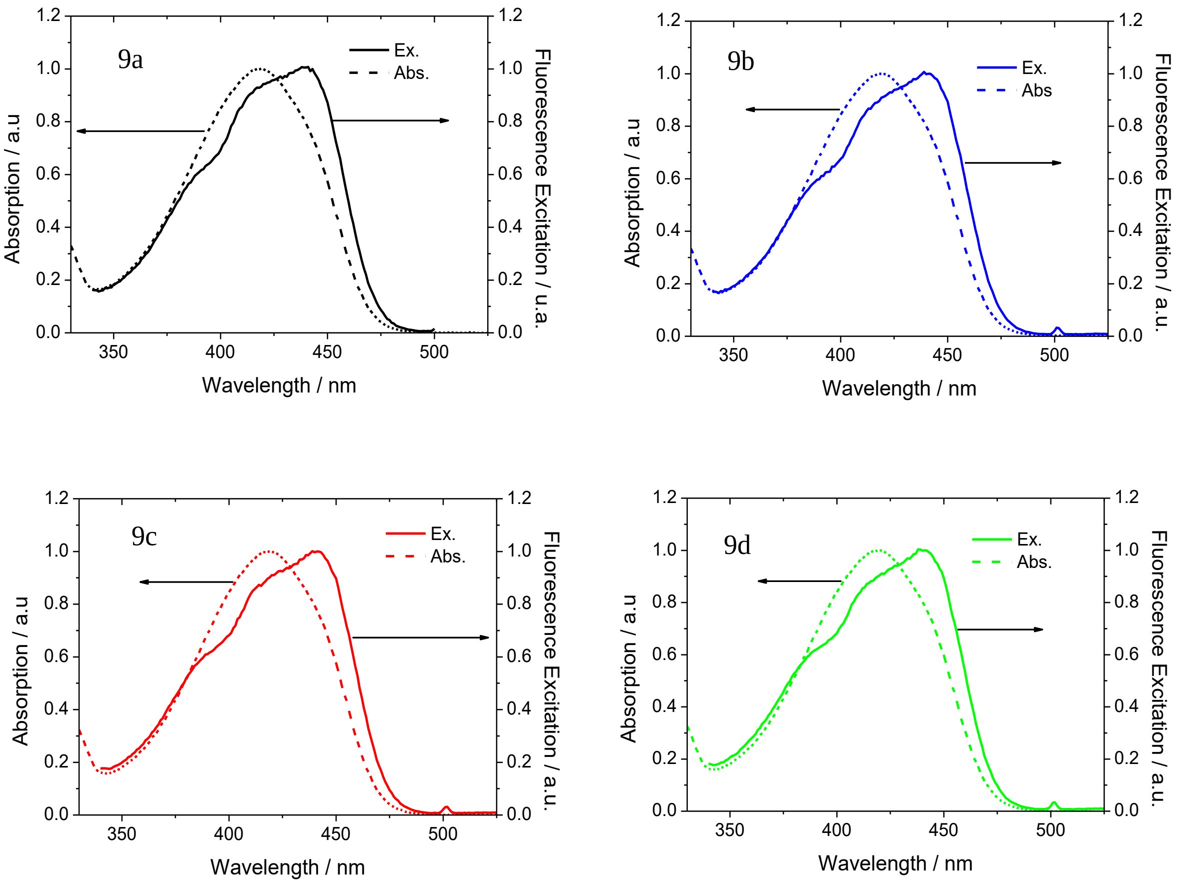

Figure 3S: Absorption (Abs) and fluorescence excitation (Ex) spectra of compounds **9a** in heptane, toluene, tetrahydrofuran (THF) and acetone solutions

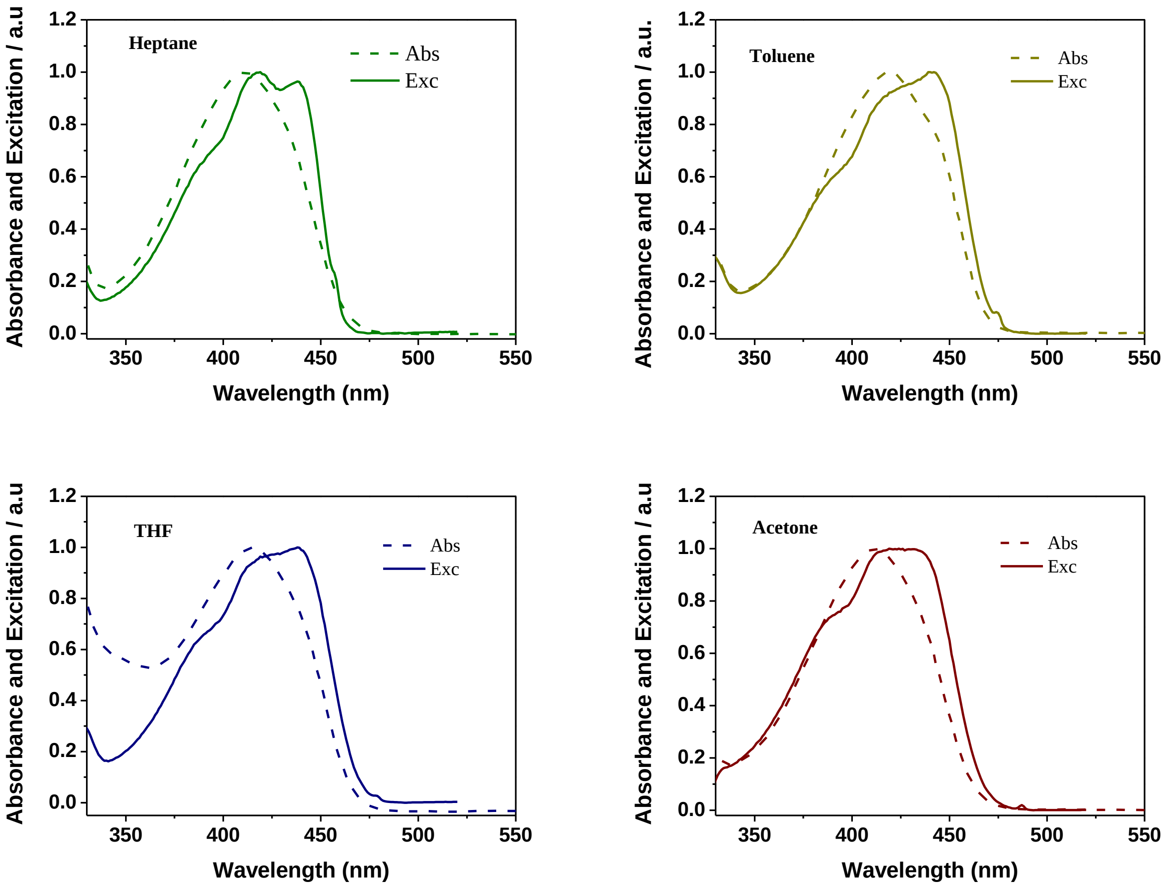

4. NMR SPECTRA:

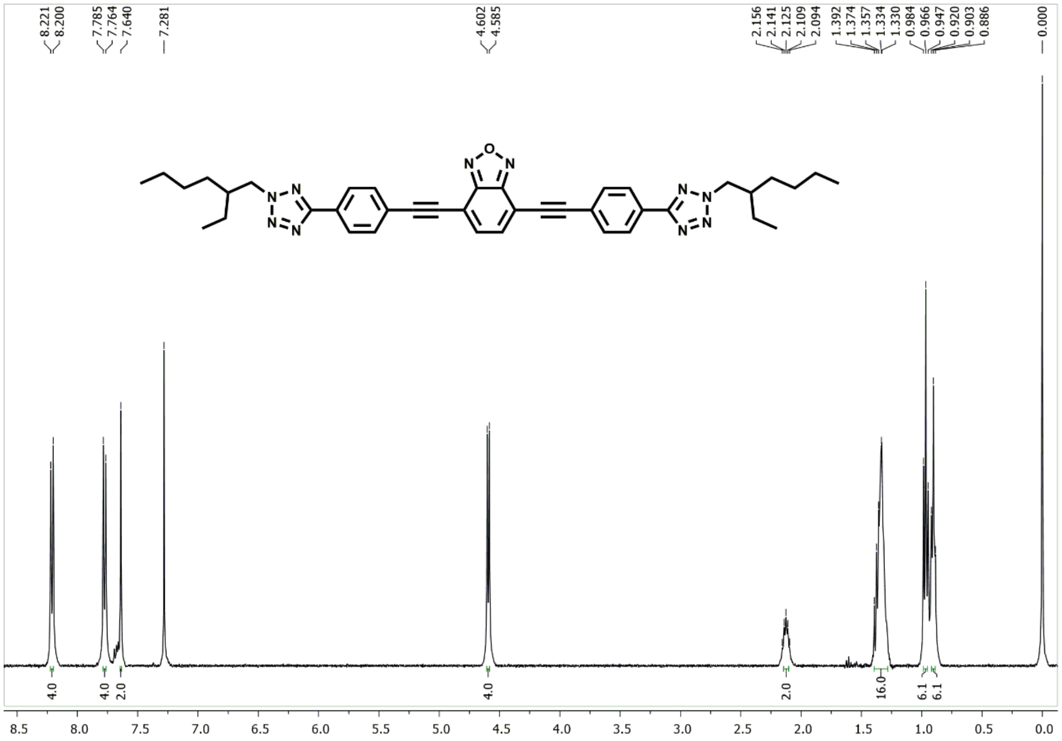

Figure 4S. ^{1}H NMR (400 MHz, $CDCl_3$) spectrum of **9a**.

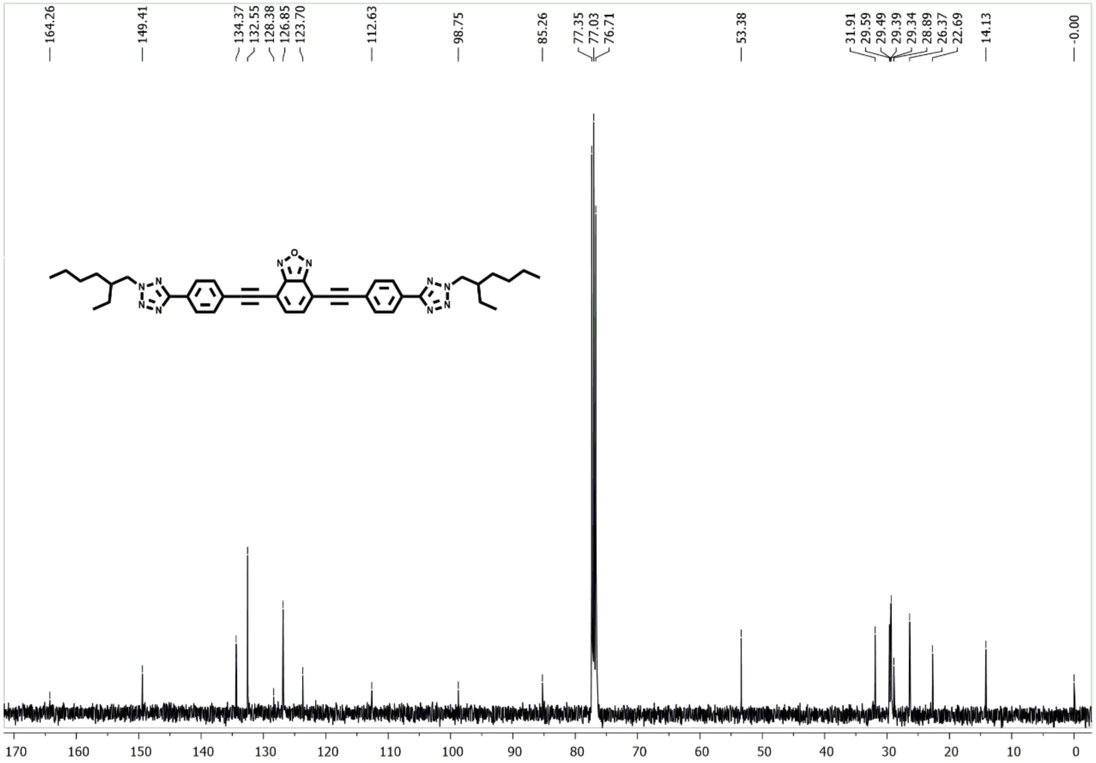

Figure 5S. ^{13}C NMR (100 MHz, $CDCl_3$) spectrum of **9a**.

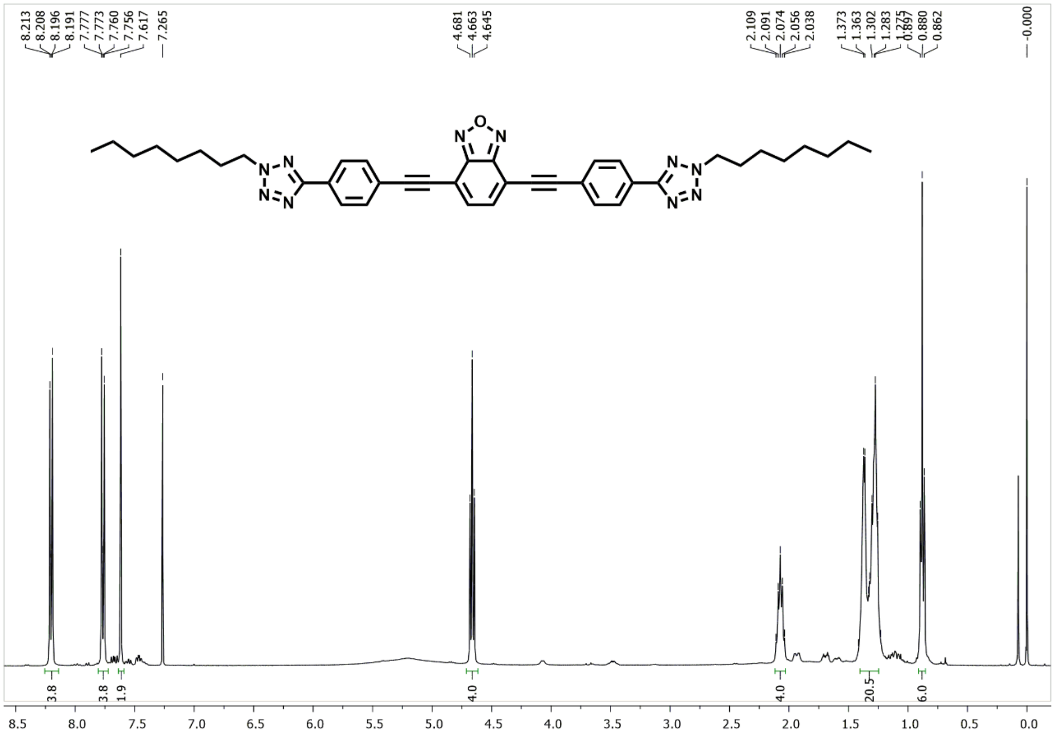

Figure 6S. ^{1}H NMR (400 MHz, $CDCl_3$) spectrum of **9b**.

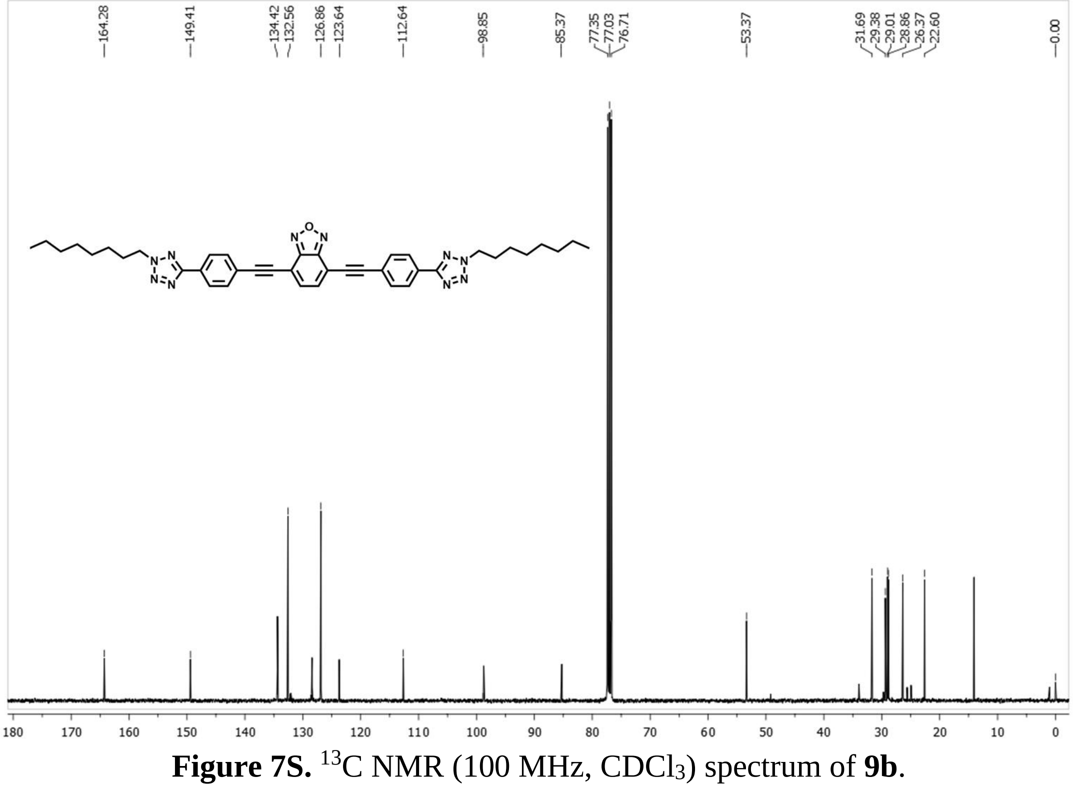

Figure 7S. ^{13}C NMR (100 MHz, $CDCl_3$) spectrum of **9b**.

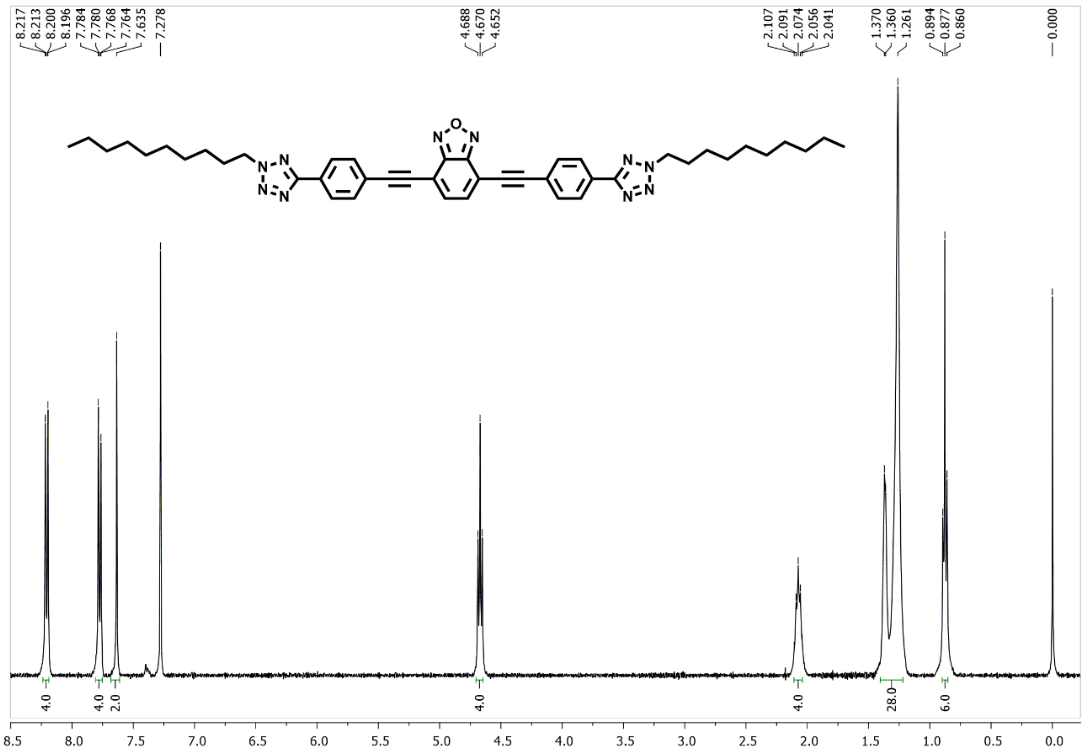

Figure 8S. ^{1}H NMR (400 MHz, $CDCl_3$) spectrum of **9c**.

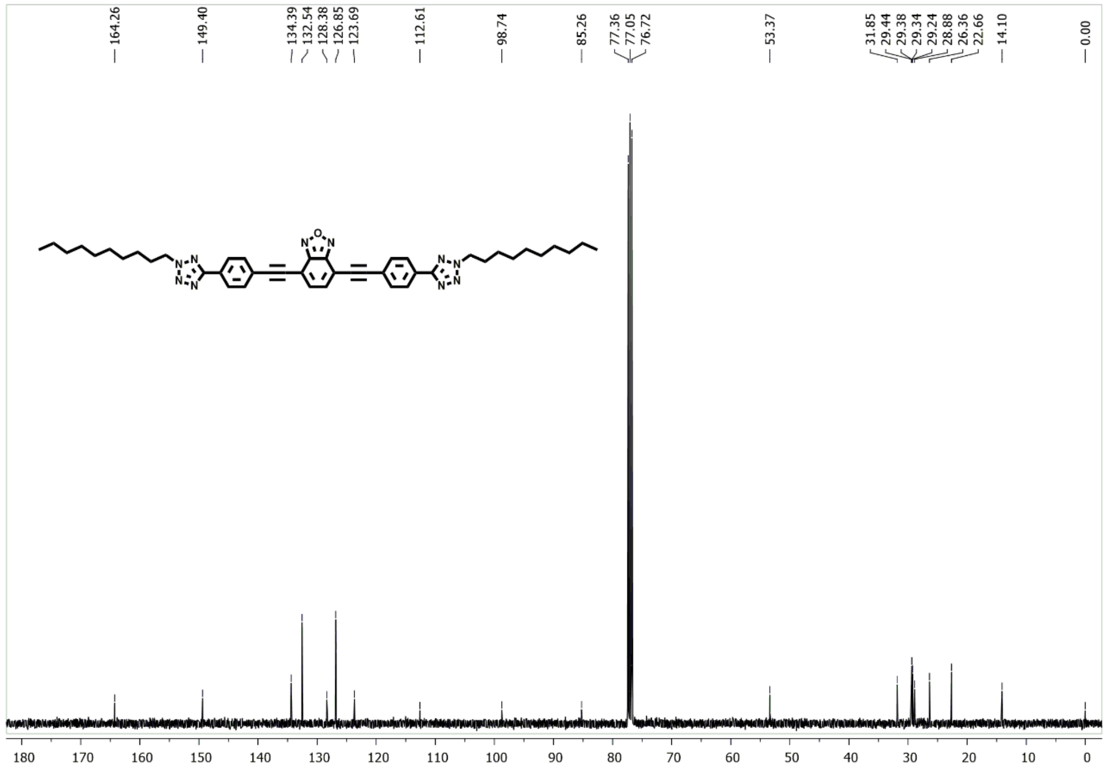

Figure 9S. ^{13}C NMR (100 MHz, $CDCl_3$) spectrum of **9c**.

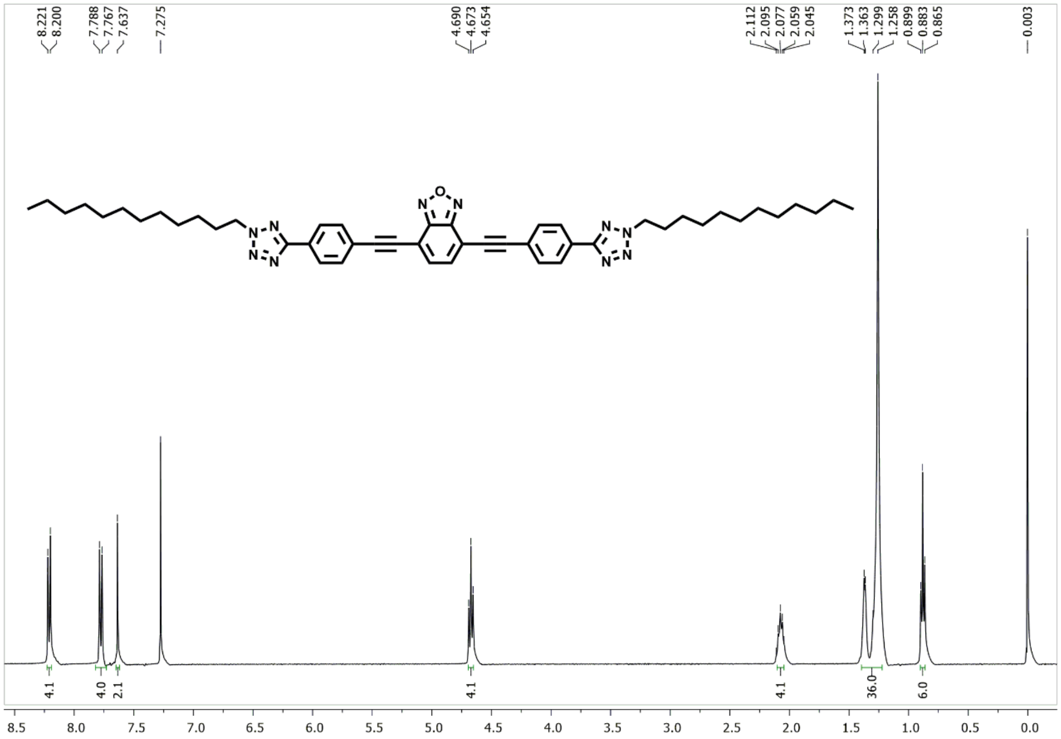

Figure 10S. ^{1}H NMR (400 MHz, $CDCl_3$) spectrum of **9d**.

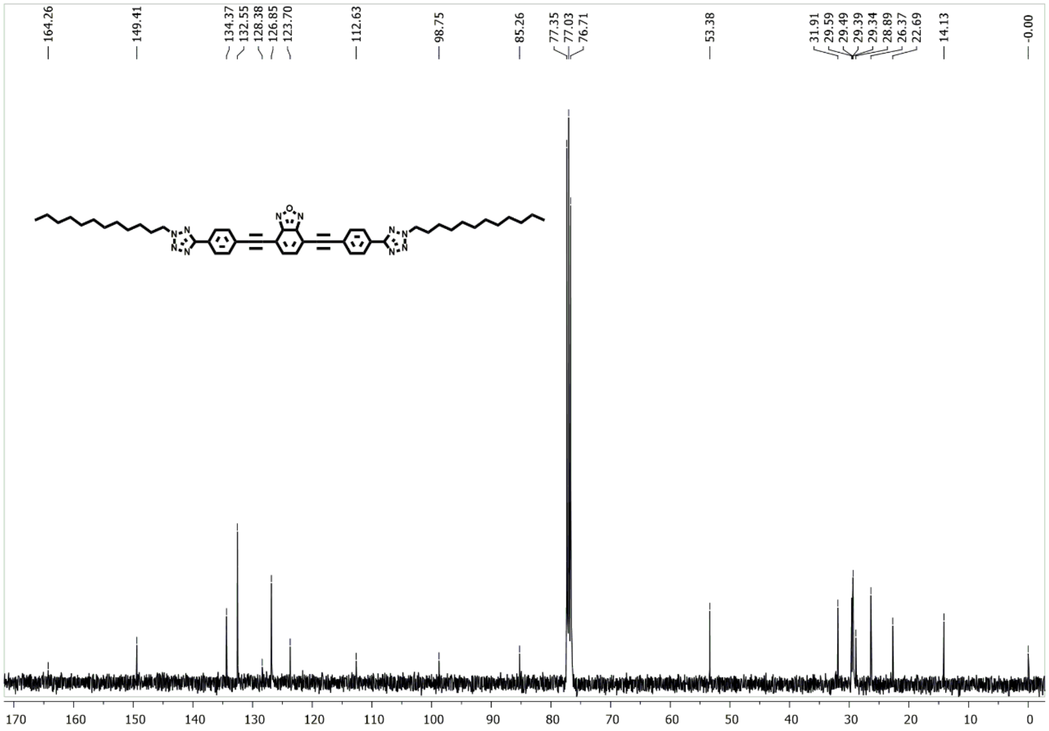

Figure 11S. ^{13}C NMR (100 MHz, $CDCl_3$) spectrum of **9d**.

5. Fourier transform infrared spectroscopy (*FTIR*)

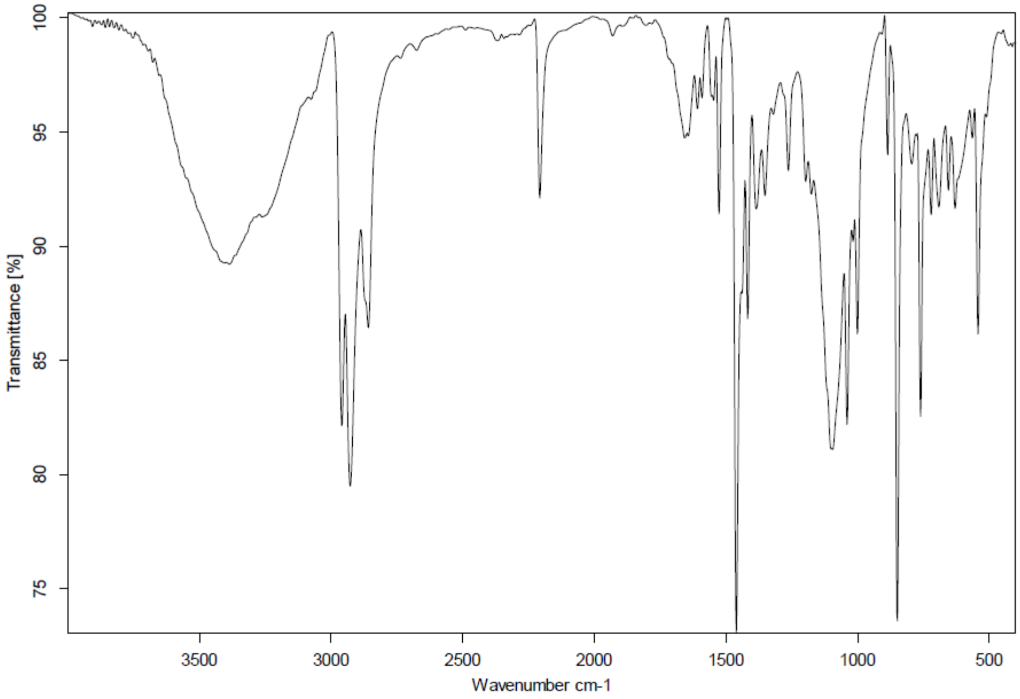

Figure 12S. The FTIR absorption spectrum (KBr disk) of **9a.**

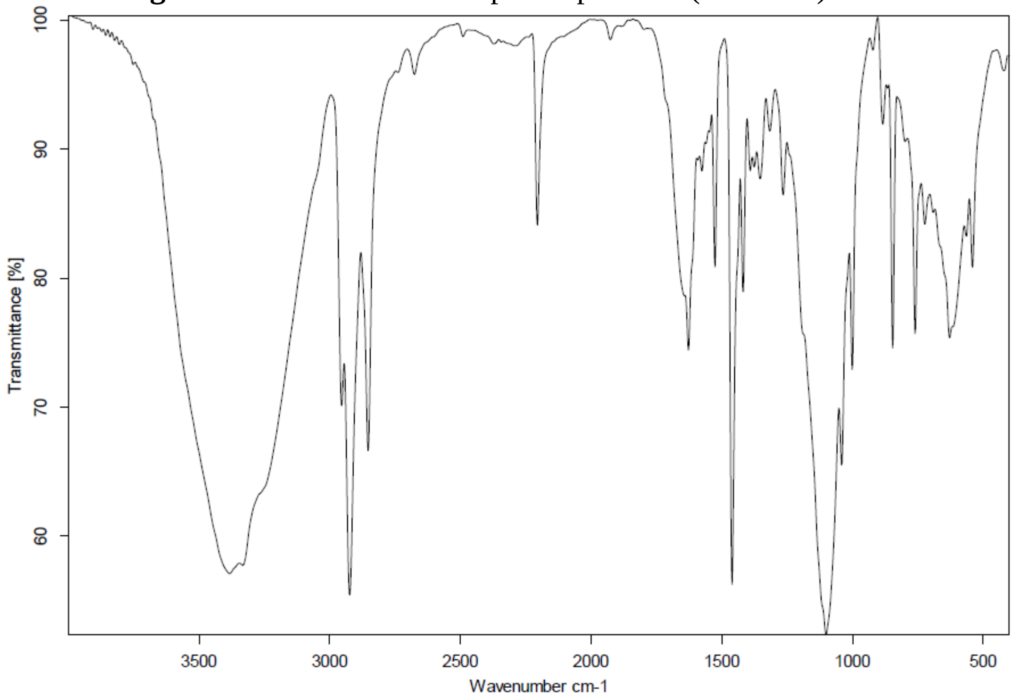

Figure 13S. The FTIR absorption spectrum (KBr disk) of **9b.**

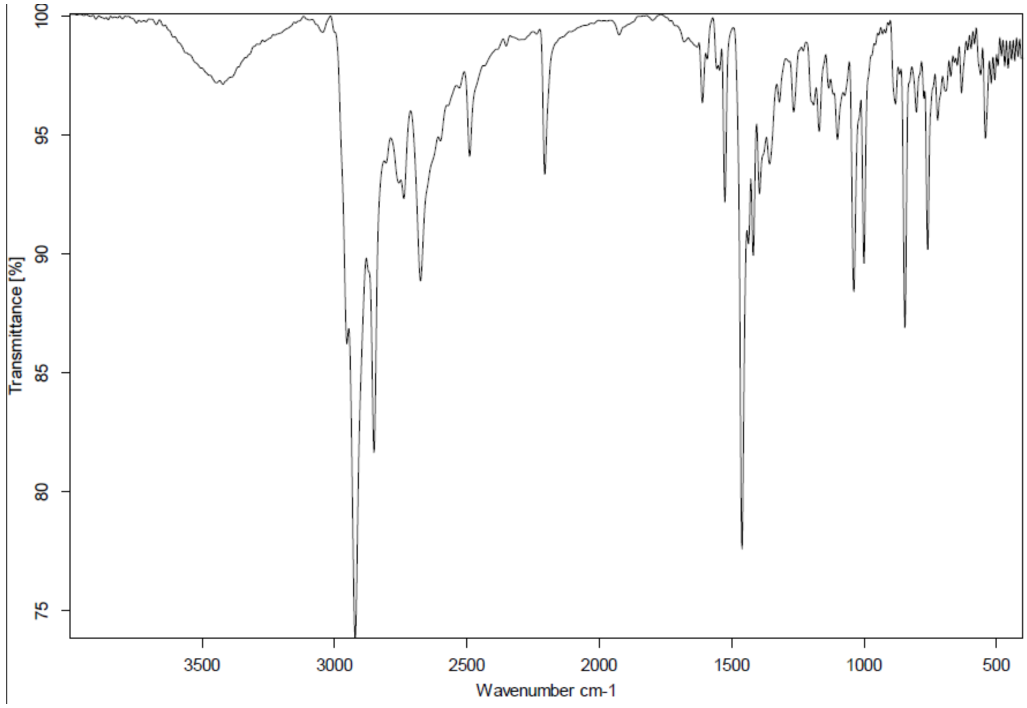

Figure 14S. The FTIR absorption spectrum (KBr disk) of **9c**.

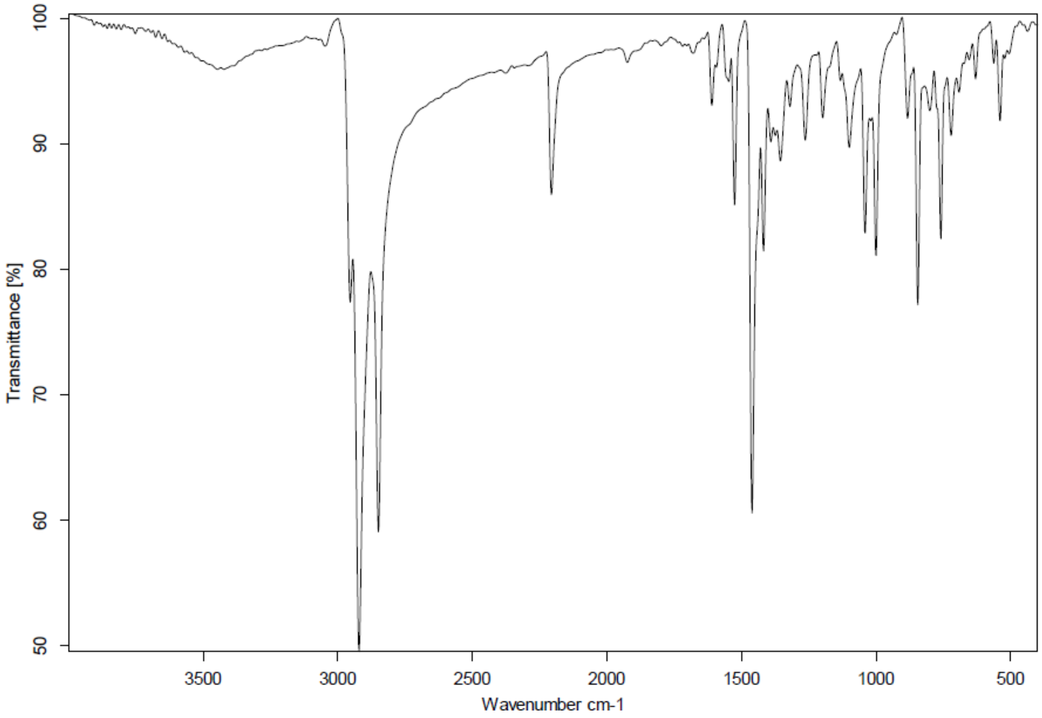

Figure 15S. The FTIR absorption spectrum (KBr disk) of **9d**.

6. HRMS:

Acquisition Parameter

Source Type	APPI	Ion Polarity	Positive	Set Nebulizer	1.5 Bar
Focus	Not active	Set Capillary	1500 V	Set Dry Heater	200 °C
Scan Begin	200 m/z	Set End Plate Offset	-500 V	Set Dry Gas	2.0 l/min
Scan End	3000 m/z	Set Collision Cell RF	700.0 Vpp	Set Divert Valve	Source

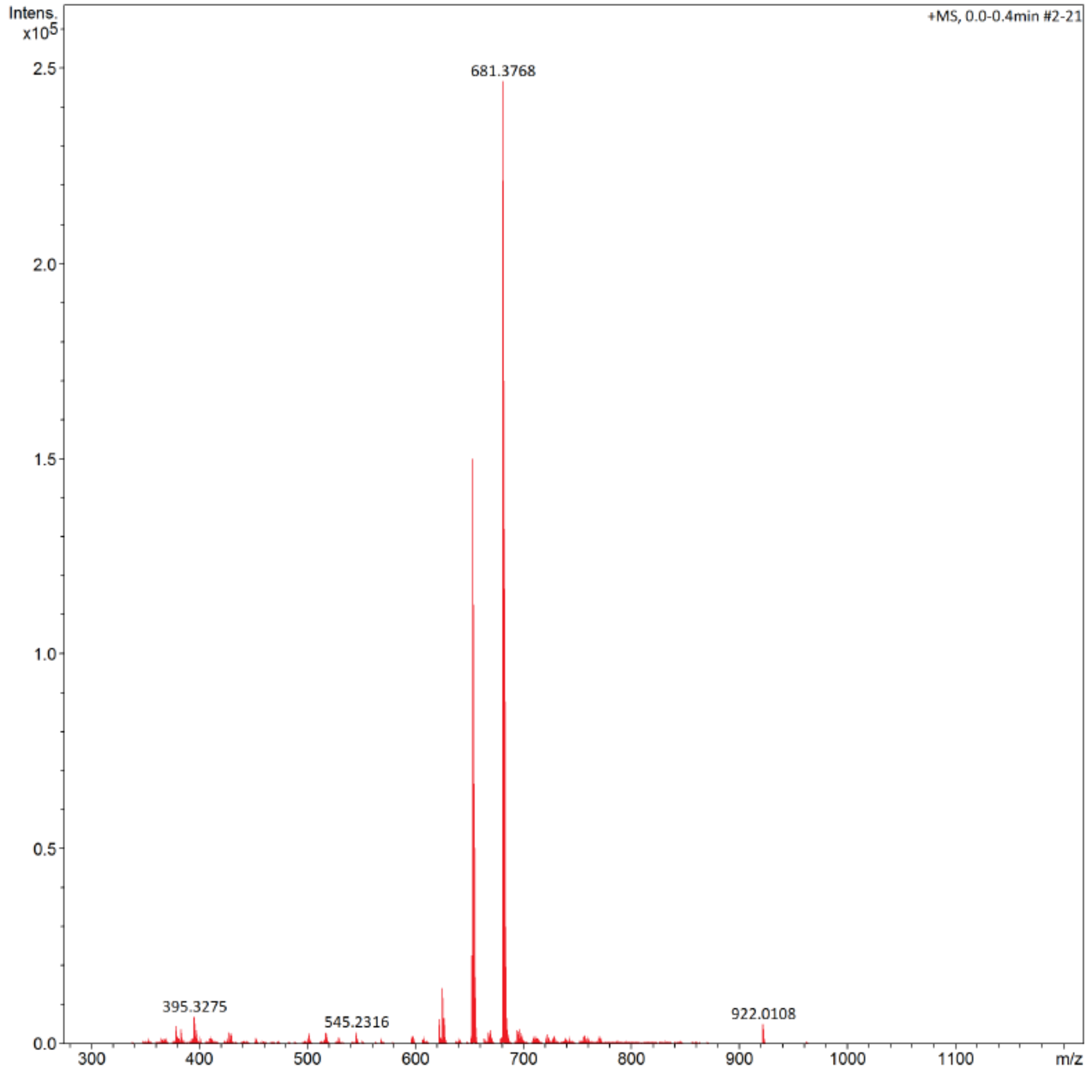

Figure 16S. High-Resolution Mass Spectrum of **9a**.

Acquisition Parameter

Source Type	APPI	Ion Polarity	Positive	Set Nebulizer	1.5 Bar
Focus	Not active	Set Capillary	1500 V	Set Dry Heater	200 °C
Scan Begin	200 m/z	Set End Plate Offset	-500 V	Set Dry Gas	2.0 l/min
Scan End	3000 m/z	Set Collision Cell RF	700.0 Vpp	Set Divert Valve	Source

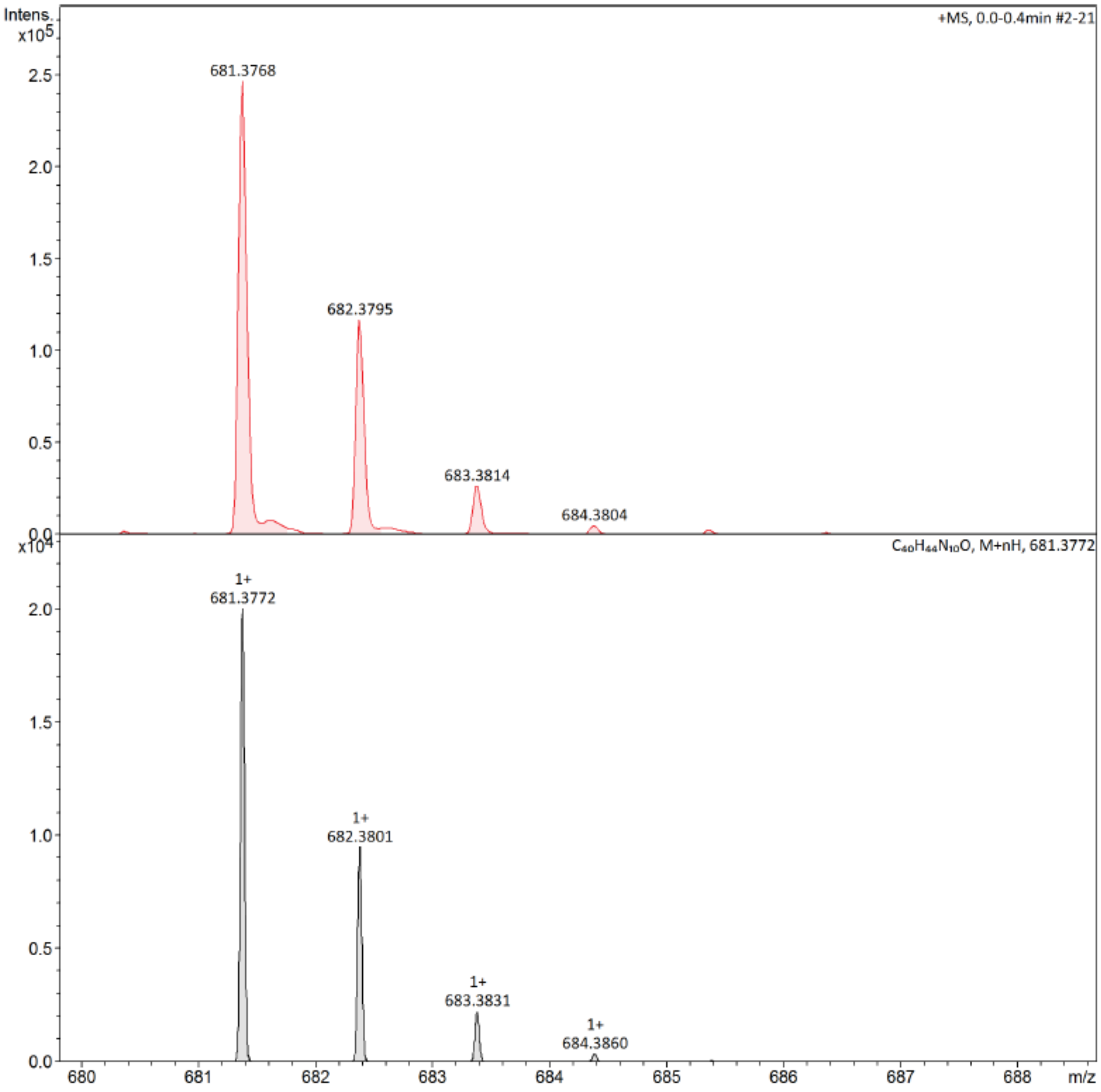

Figure 17S. Isotope Distribution Mass Spectrum of **9a**.

Acquisition Parameter

Source Type	APPI	Ion Polarity	Positive	Set Nebulizer	1.5 Bar
Focus	Not active	Set Capillary	1500 V	Set Dry Heater	200 °C
Scan Begin	200 m/z	Set End Plate Offset	-500 V	Set Dry Gas	2.0 l/min
Scan End	3000 m/z	Set Collision Cell RF	700.0 Vpp	Set Divert Valve	Source

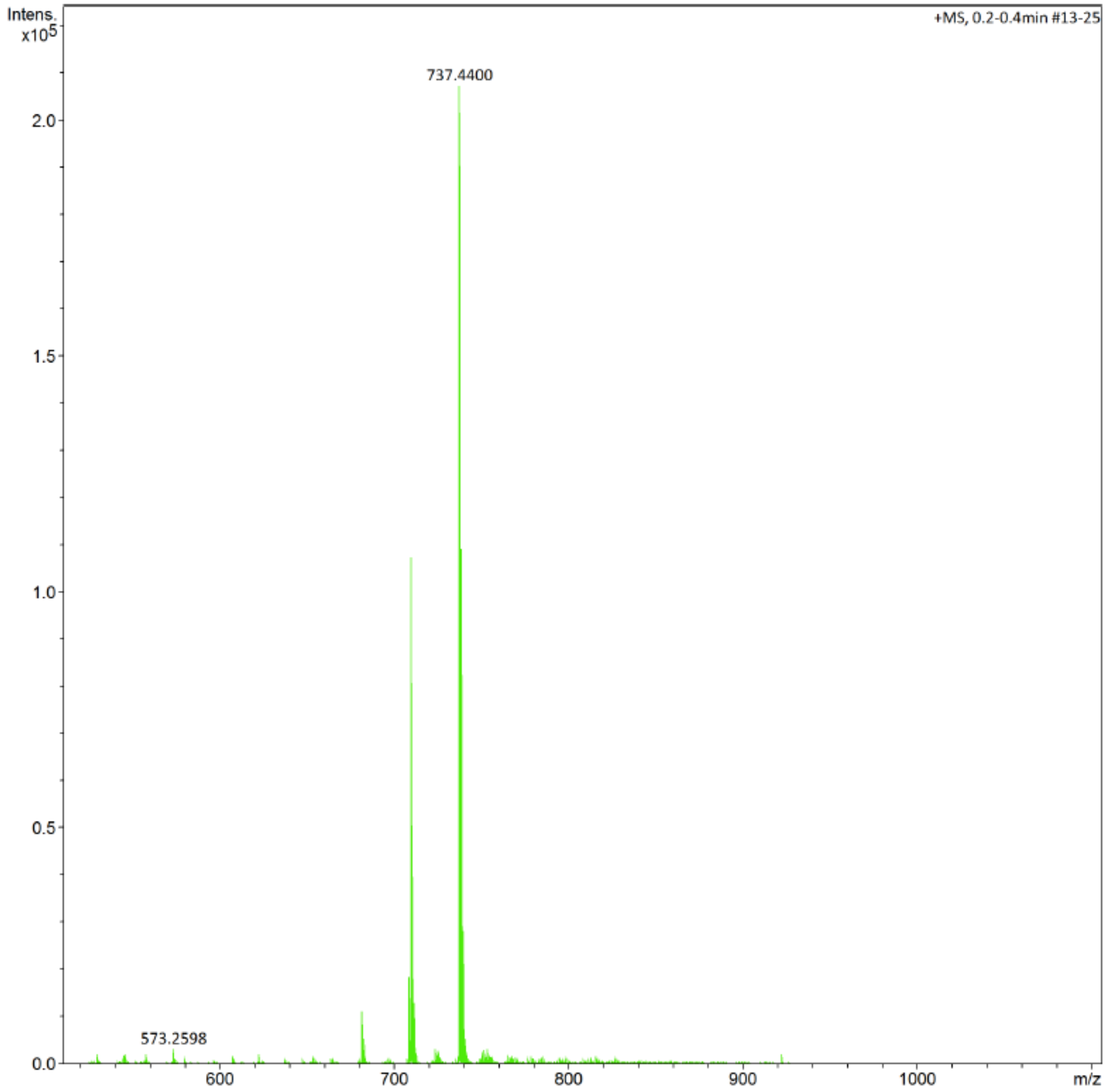

Figure 18S. High-Resolution Mass Spectrum of **9b**.

Acquisition Parameter

Source Type	APPI	Ion Polarity	Positive	Set Nebulizer	1.5 Bar
Focus	Not active	Set Capillary	1500 V	Set Dry Heater	200 °C
Scan Begin	200 m/z	Set End Plate Offset	-500 V	Set Dry Gas	2.0 l/min
Scan End	3000 m/z	Set Collision Cell RF	700.0 Vpp	Set Divert Valve	Source

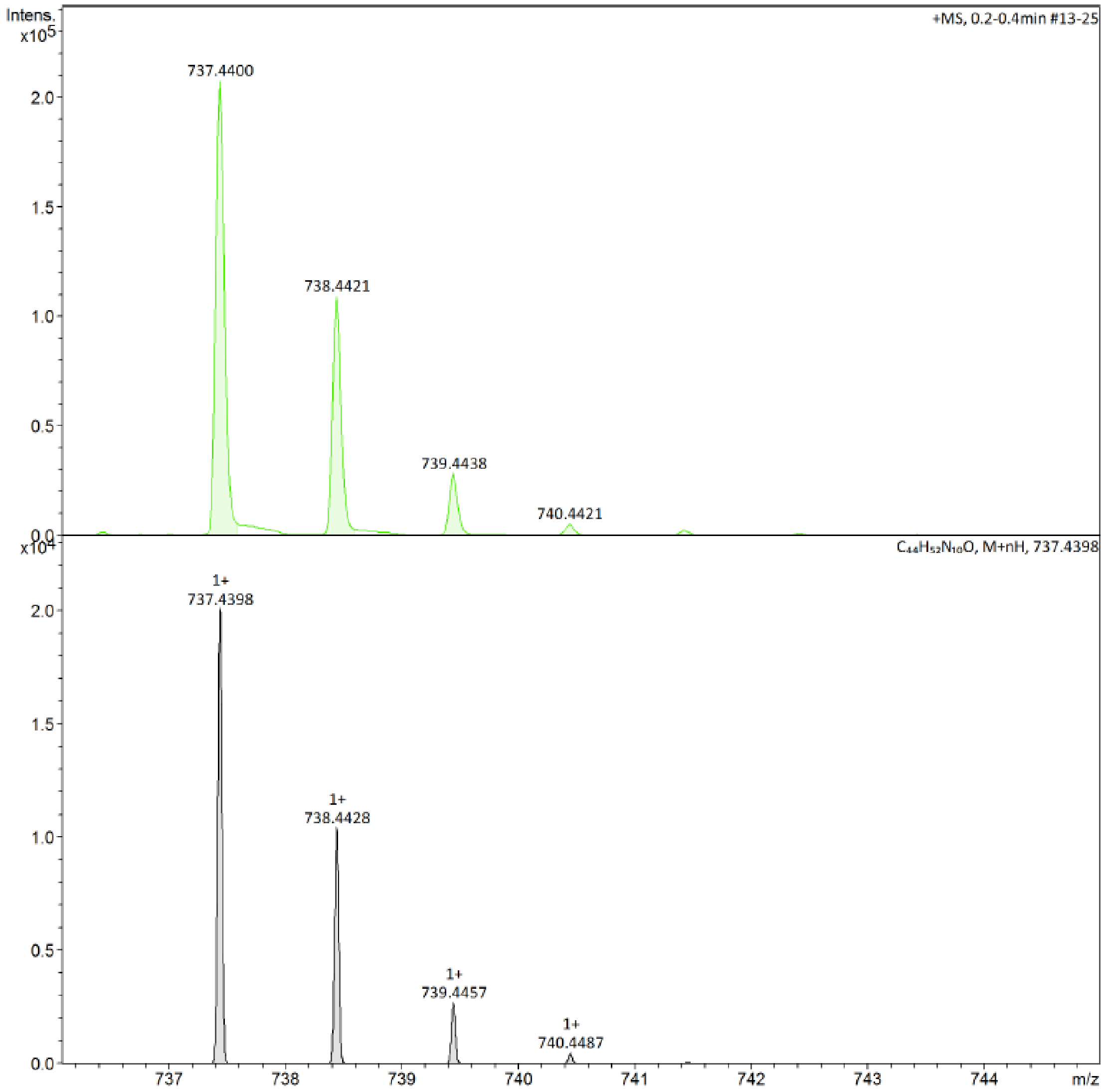

Figure 19S. Isotope Distribution Mass Spectrum of **9b**.

Acquisition Parameter

Source Type	APPI	Ion Polarity	Positive	Set Nebulizer	1.5 Bar
Focus	Not active	Set Capillary	1500 V	Set Dry Heater	200 °C
Scan Begin	200 m/z	Set End Plate Offset	-500 V	Set Dry Gas	2.0 l/min
Scan End	3000 m/z	Set Collision Cell RF	700.0 Vpp	Set Divert Valve	Source

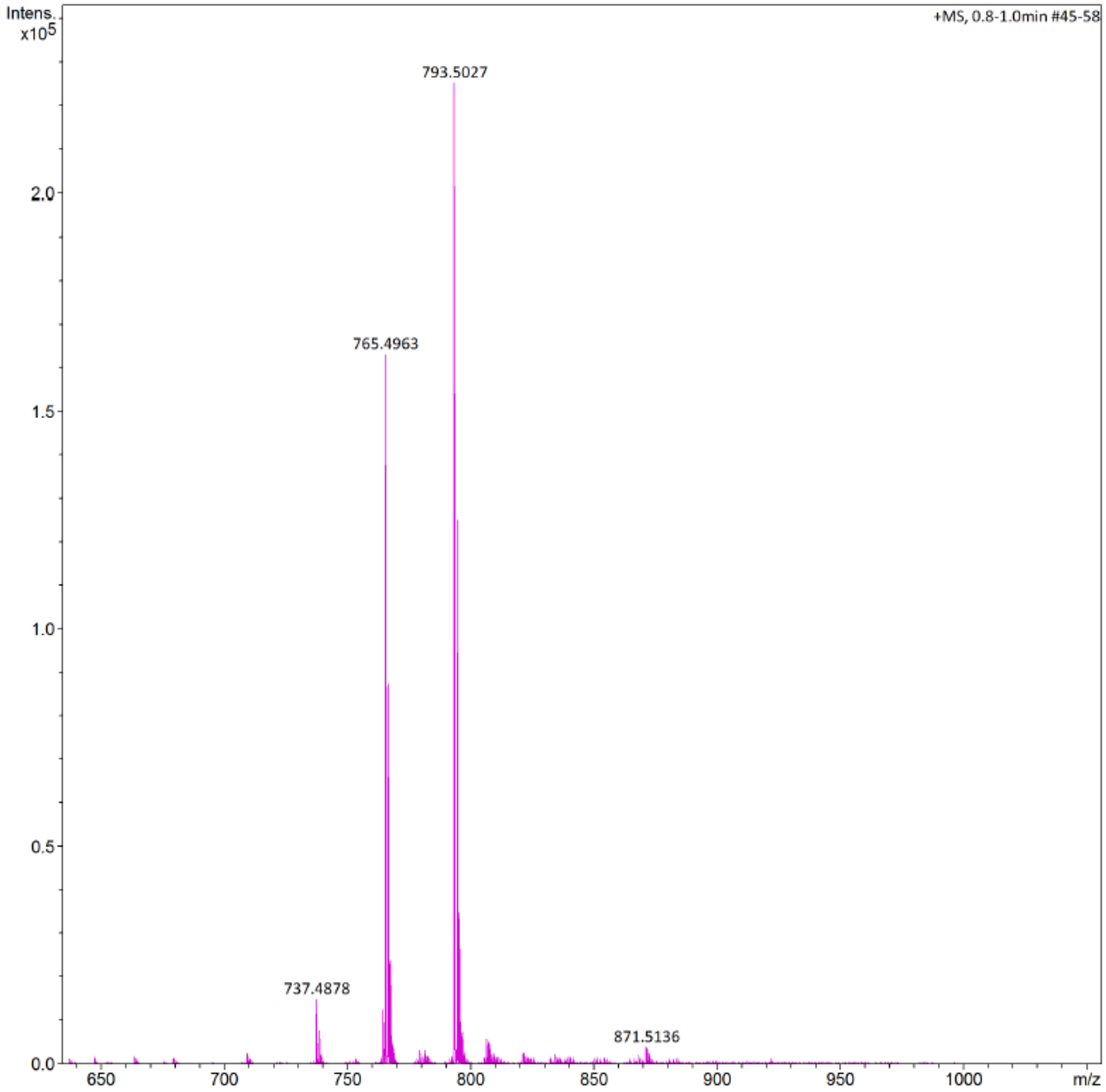

Figure 20S. High-Resolution Mass Spectrum of **9c**.

Acquisition Parameter

Source Type	APPI	Ion Polarity	Positive	Set Nebulizer	1.5 Bar
Focus	Not active	Set Capillary	1500 V	Set Dry Heater	200 °C
Scan Begin	200 m/z	Set End Plate Offset	-500 V	Set Dry Gas	2.0 l/min
Scan End	3000 m/z	Set Collision Cell RF	700.0 Vpp	Set Divert Valve	Source

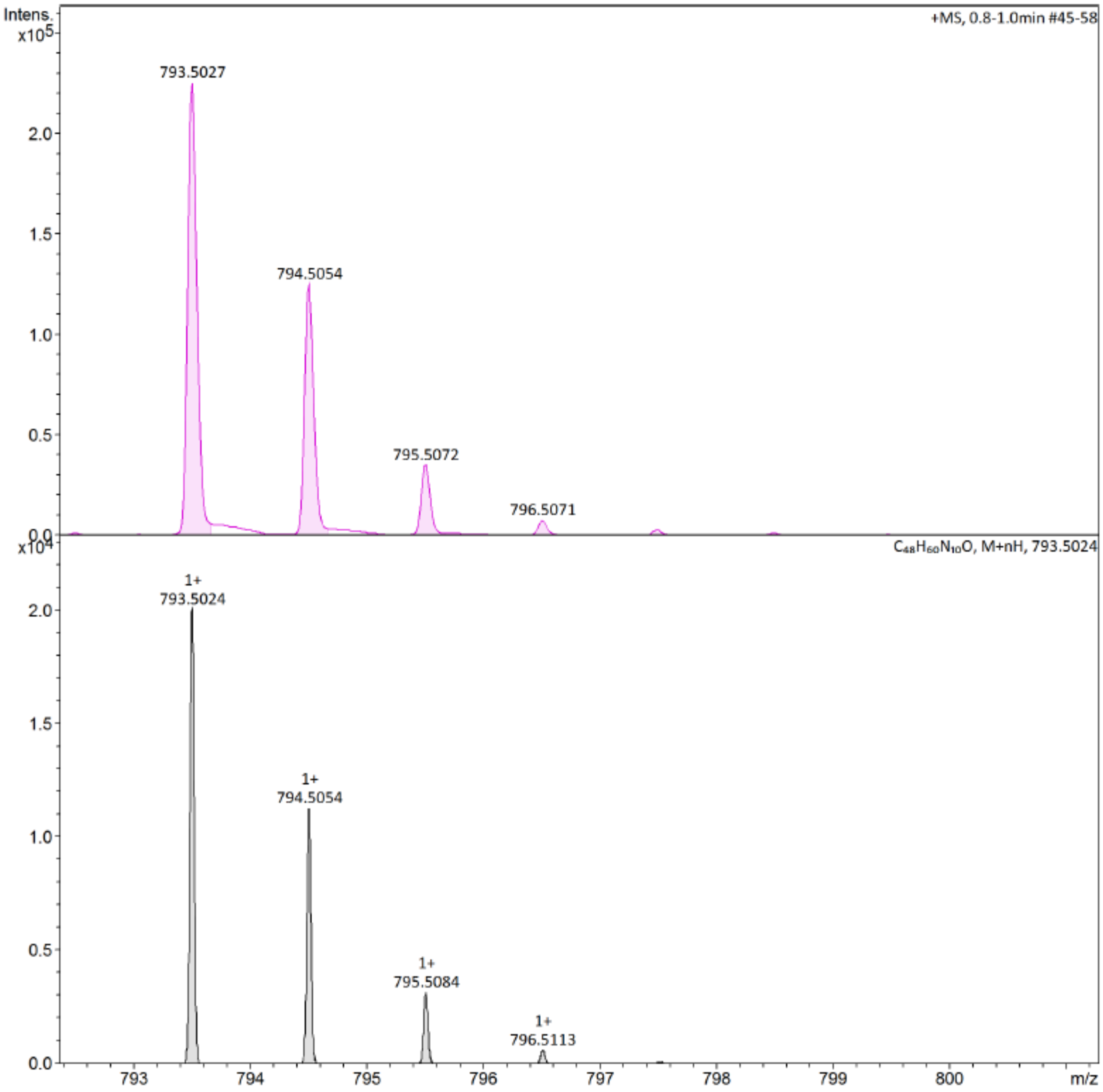

Figure 21S. Isotope Distribution Mass Spectrum of **9c**.

Acquisition Parameter

Source Type	APPI	Ion Polarity	Positive	Set Nebulizer	1.5 Bar
Focus	Not active	Set Capillary	1500 V	Set Dry Heater	200 °C
Scan Begin	200 m/z	Set End Plate Offset	-500 V	Set Dry Gas	2.0 l/min
Scan End	3000 m/z	Set Collision Cell RF	600.0 Vpp	Set Divert Valve	Source

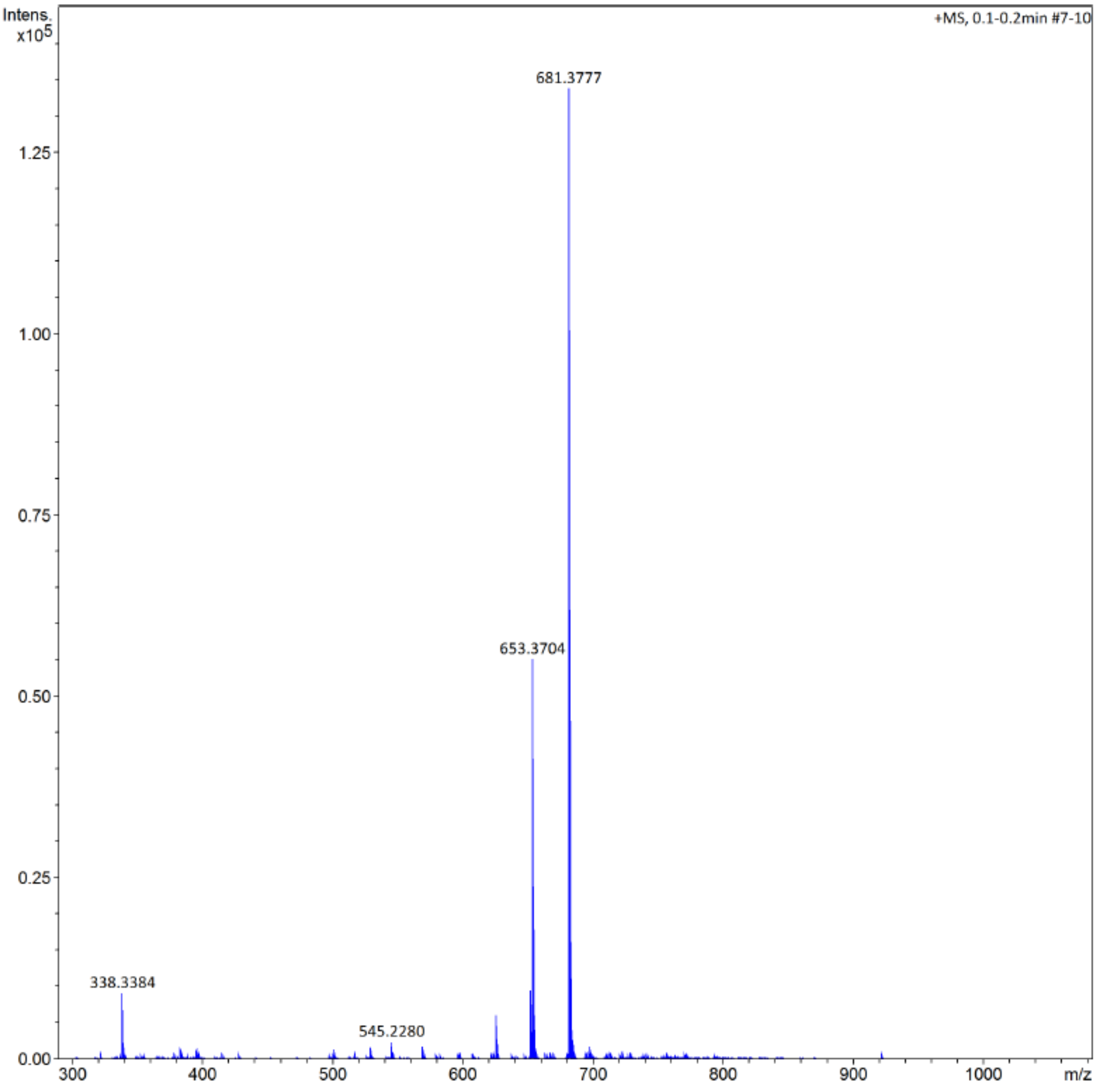

Figure 22S. High-Resolution Mass Spectrum of **9d**.

Acquisition Parameter

Source Type	APPI	Ion Polarity	Positive	Set Nebulizer	1.5 Bar
Focus	Not active	Set Capillary	1500 V	Set Dry Heater	200 °C
Scan Begin	200 m/z	Set End Plate Offset	-500 V	Set Dry Gas	2.0 l/min
Scan End	3000 m/z	Set Collision Cell RF	600.0 Vpp	Set Divert Valve	Source

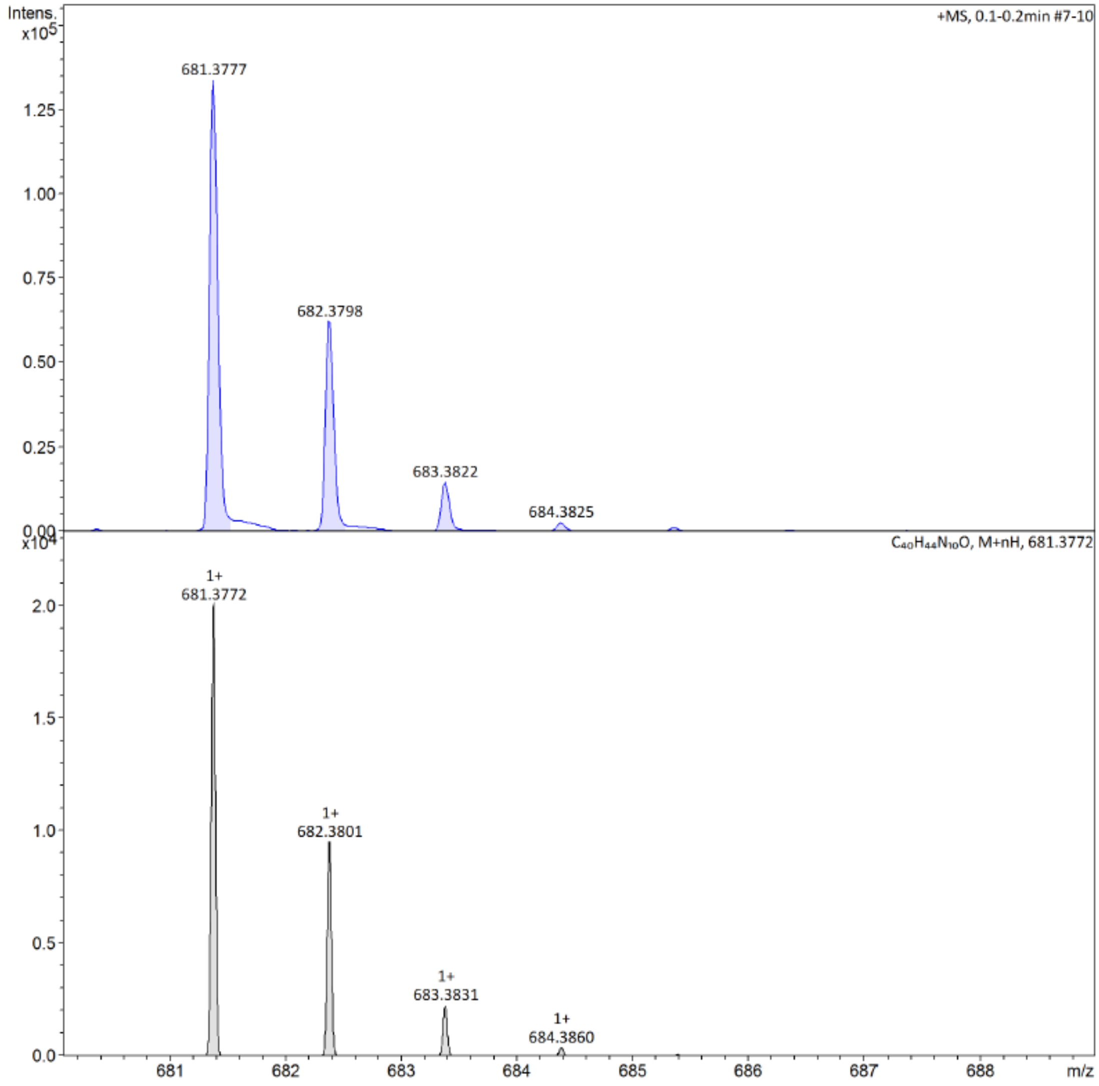

Figure 23S. Isotope Distribution Mass Spectrum of **9d**.
